# An at-home blood collection device for remote immune monitoring by high-parameter flow cytometry

**DOI:** 10.1172/jci.insight.201116

**Published:** 2026-04-08

**Authors:** Andrew J. Konecny, Fang Yun Lim, Eva Domenjo-Vila, Erika Lovas, Rachel L. Blazevic, Louise E. Kimball, Michael Boeckh, Alpana Waghmare, Martin Prlic

**Affiliations:** 1 Vaccine and Infectious Disease Division, Fred Hutchinson Cancer Center, Seattle, Washington, USA.; 2 Department of Immunology,; 3 Department of Medicine, and; 4 Department of Pediatrics, University of Washington, Seattle, Washington, USA.; 5 Seattle Children’s Research Institute, Seattle, Washington, USA.

**Keywords:** Cell biology, Clinical Research, Immunology, Health services research

## Abstract

At-home blood collection devices (ABCDs) can facilitate study participation for remote and rural cohorts. Previous studies used ABCDs to interrogate samples by proteomics and sequencing approaches. We wanted to address the question of whether this approach could be used to assess live immune cells with high-parameter flow cytometry to enable remote immune monitoring. We first compared blood from standard venipuncture with ABCD blood draws, followed by assessment of the impact of sample shipping on immune cell viability and phenotyping. We found that capillary blood collected with a Tasso+ device and concurrently drawn venipuncture blood samples had highly congruent immune cell composition and phenotype. Shipment of Tasso+ samples via the United States Postal Service altered the myeloid compartment, but T cell numbers, subsets, and phenotypes remained remarkably stable compared with non-shipped samples. Finally, we describe a flow cytometry analysis framework that allowed for direct sample comparison even when samples were stained and analyzed over a time period of 1.5 years. Overall, our data highlight the feasibility of using ABCDs combined with subsequent flow cytometry analysis for remote immune monitoring. Additionally, our study also identifies areas that could be improved to further promote the use of ABCDs for immune monitoring.

## Abstract 2

In our study we demonstrate the feasibility of an at home blood collection device for remote immune monitoring by high parameter flow cytometry.

## Introduction

Analysis of peripheral blood immune cells by flow cytometry is routinely used as a readout in a wide range of clinical trials including vaccine trials and cancer therapy trials (1–3). Moreover, peripheral blood samples have been used to assess the immune response to infections, including hundreds of COVID-19–related studies (4–6), and to enable longitudinal immune monitoring of transplant patients and other patients with compromised immune systems (7). Peripheral blood is typically drawn by a phlebotomist or clinical research coordinator, thus often requiring studies to be centralized at a clinic or research institute. Travel to these centers for blood draws is a barrier for a wide range of potential participants, including individuals who live in remote and rural communities (8). For example, in the United States, there is a decrease in indigenous populations living near centers performing clinical trials (9) and an underrepresentation of indigenous populations in clinical trial studies (10). In decentralized studies, individuals can participate outside of a center, including in their own home. Thus, decentralized studies can increase the radius of recruitment from research centers, which allows for the recruited cohort to better represent a population’s true demographics (11). Decentralized studies could be beneficial for clinical care in areas that have high incidence of cancer, pulmonary disease, and diabetes but lack easily accessible clinical centers (12). Overall, decentralized studies allow individuals to participate in clinical studies from the convenience of their home, and thus have the potential to increase participation and retention rates, and to facilitate longitudinal sampling (8, 13–15).

At-home blood collection devices (ABCDs) facilitate decentralized studies because they remove the requirement of travel to a clinic or clinical resources such as trained professionals to draw blood. Briefly, most ABCDs, including the Tasso+ device used in our study, typically collect capillary blood using a lancet mechanism (16). These have been used successfully for the collection of blood in numerous studies including pharmacokinetic (17, 18), viral load (19), antibody/antigen (20), bulk transcriptomics (21), and bulk proteomic studies (22, 23). To the best of our knowledge, the feasibility of using ABCDs for subsequent live cell analysis has yet to be tested.

Here, we report (a) immunological congruence between venous and capillary (using the Tasso+ blood collection device) blood draws, (b) Tasso+ blood draws yield a sufficient number of cells to allow for immune cell subsetting, (c) cells had greater than 75% viability if samples were returned to the central lab via non–temperature-regulated courier services within 3 days after collection, and (d) T cell subset distribution and phenotypes were stable despite using EDTA-coated tubes without any additional preservation or stabilization buffers. Our study also highlights areas for improvement. We found that different blood-processing methods introduce substantial technical variability, and that stabilization buffers will be needed to improve myeloid cell viability. Separately, to ensure that longitudinal studies are indeed technically feasible, we tested a standard operating procedure to assess suitability for flow cytometry analysis with minimal variability over time. We report that a set of biological replicates consisting of PBMCs processed from a single leukapheresis sample could be stained with antibodies and analyzed by flow cytometry repeatedly over the course of 18 months and yielded highly consistent data. Overall, our study provides much needed proof-of-principle data to extend the use of ABCDs for immune cell analysis via high-parameter flow cytometry.

## Results

### The immune cell populations in capillary blood closely resemble those collected by venipuncture.

**Figure 1:**
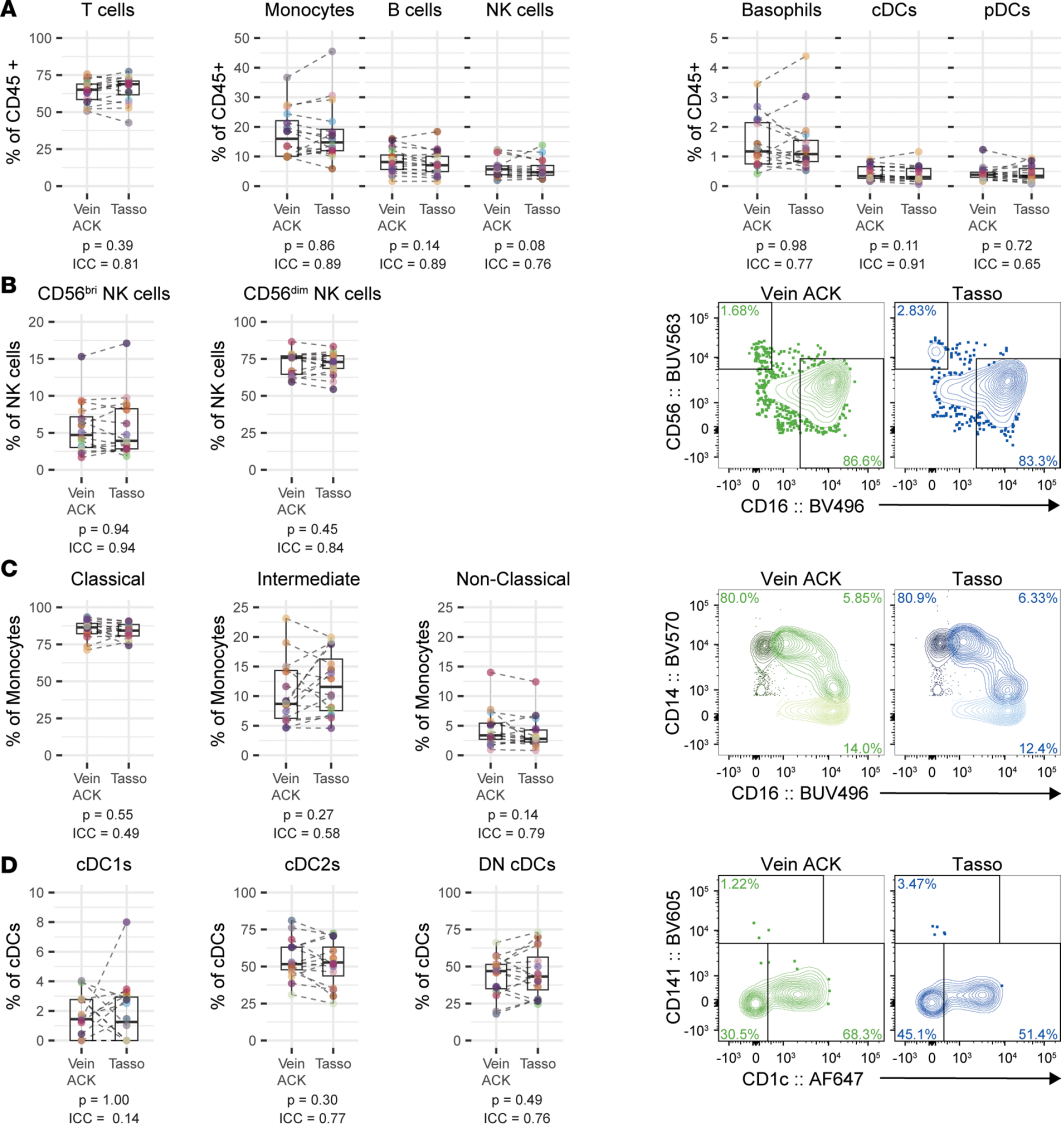
Capillary blood closely resembles venous blood. (**A**) Quantification of major immune cell subsets as frequencies of CD45^+^ non-neutrophils, comparing venipuncture-collected ACK-lysed whole blood (Vein ACK) and ACK-lysed whole blood collected by Tasso+ (Tasso) (*n* = 16). (**B**–**D**) Quantification and representative plots of NK cell subsets (**B**), monocyte subsets (**C**), and cDC subsets (**D**) comparing Vein ACK and Tasso samples (*n* = 16). Data shown are from 2 independent experiments. Statistical analyses were performed using Wilcoxon’s signed-rank test and intraclass correlation coefficient test.

We first wanted to determine whether blood from the Tasso+ recapitulates blood collected via venipuncture for immunological profiling of cell subsets by comparing donor- and visit-matched venipuncture and Tasso+ samples collected from healthy volunteers. Most immunological studies use PBMCs, which are generated by running blood obtained via venipuncture over a Ficoll density gradient to deplete RBCs and polymorphonuclear cells. The blood volume that we collected from Tasso+ devices ranged from 100 to 1,000 μL, which is not suitable for processing by a Ficoll gradient. Thus, we tested for 2 variables in 2 sets of experiments: capillary blood (blood collected with Tasso+) versus venipuncture blood, and ACK lysis of RBCs versus Ficoll gradient. First, we compared venipuncture to Tasso+ blood using ACK to lyse RBCs in both samples. Up to 1 mL of blood from either collection method was processed by ACK lysis, then processed for cryopreservation and stored in liquid nitrogen until analysis. For analysis, samples were then split into 2 equal aliquots for staining with 2 high-dimensional flow cytometry panels to assess the antigen-presenting cell (APC) and T cell compartments (Supplemental Table 1; supplemental material available online with this article; https://doi.org/10.1172/jci.insight.201116DS1) (24, 25). Briefly, our APC panel focused on delineating monocyte and conventional DC (cDC) subsets in addition to plasmacytoid DCs (pDCs), basophils, NK cells and their subsets, total B cells, and total T cells (Supplemental Figure 1A). The panel contained 17 additional markers for continued subsetting and to inform APC’s function, trafficking, and costimulatory and inhibitory capacity to activate. The T cell panel further dissected T cells into conventional CD4^+^ T cells, conventional CD8^+^ T cells, γδ T (TCRgd T) cells, mucosal-associated invariant T (MAIT) cells, and Treg cells in addition to their memory subsets (Supplemental Figure 1B). B cells in this panel can be further subdivided into memory B cells, naive B cells, and plasmablasts (Supplemental Figure 1C). The T cell panel contained 18 markers to inform activation status, cytotoxicity, residency, and inhibition. We observed no change in the population frequency distribution of the innate immune compartment consisting of monocytes, cDCs, pDCs, basophils, or NK cells when comparing venipuncture with capillary blood. We additionally observed no change in the adaptive immune compartment consisting of T cells and B cells (Figure 1A). Further subsetting of NK cells into those that have high cytokine production (CD56^hi^CD16^–^) and those that have higher cytotoxic potential (CD56^lo^CD16^+^) revealed no difference in the composition of the NK cell pool (Figure 1B). This observation was also true for monocytes when divided into their constituent subsets: classical monocytes (CD14^+^CD16^–^), intermediate monocytes (CD14^+^CD16^+^), and nonclassical monocytes (CD14^lo^CD16^+^) (Figure 1C). cDCs can be further divided into the cross-presenting cDC1s (CD141^+^), cDC2s (CD1c^+^), and double-negative cDCs (CD141^–^CD1c^–^). In our cohort, we did not observe changes in the cDC compartment; however, the low cell numbers of cDC1s obtained with low-volume input samples likely made it difficult to accurately test for a difference between the 2 sampling procedures (Figure 1D). When further subdividing the T cell compartment, we found no differences in the relative frequency of conventional CD4^+^ T cells, conventional CD8^+^ T cells, TCRgd T cells, or MAIT cells. We did observe a minimal (0.52%) but statistically significant increase of Treg cells in capillary blood when compared with venous blood (Supplemental Figure 2A). Additionally, subsetting of the B cell compartment did reveal modest yet statistically significant changes in memory B cells, naive B cells, and plasmablasts (Supplemental Figure 2B).

We next assessed the intraclass correlation coefficient (ICC) as a measurement of reproducibility. ICC values between 0 and 0.49 are considered to be poor, 0.5–0.74 are moderate, 0.75–0.89 are good, and 0.9–1 are excellent in terms of agreement (26). Most immune subsets with an insignificant *P* value for pairwise testing had an ICC value that indicated good to excellent reproducibility (ICC > 0.75) (Figure 1 and Supplemental Figure 2). Together, these data suggest that the immune cell populations obtained from capillary blood using a Tasso+ device are essentially congruent to venipuncture blood when assessed in depth by 2 high-parameter flow cytometry panels.

### Composition and phenotype of immune cells vary with different blood-processing protocols.

**Figure 2:**
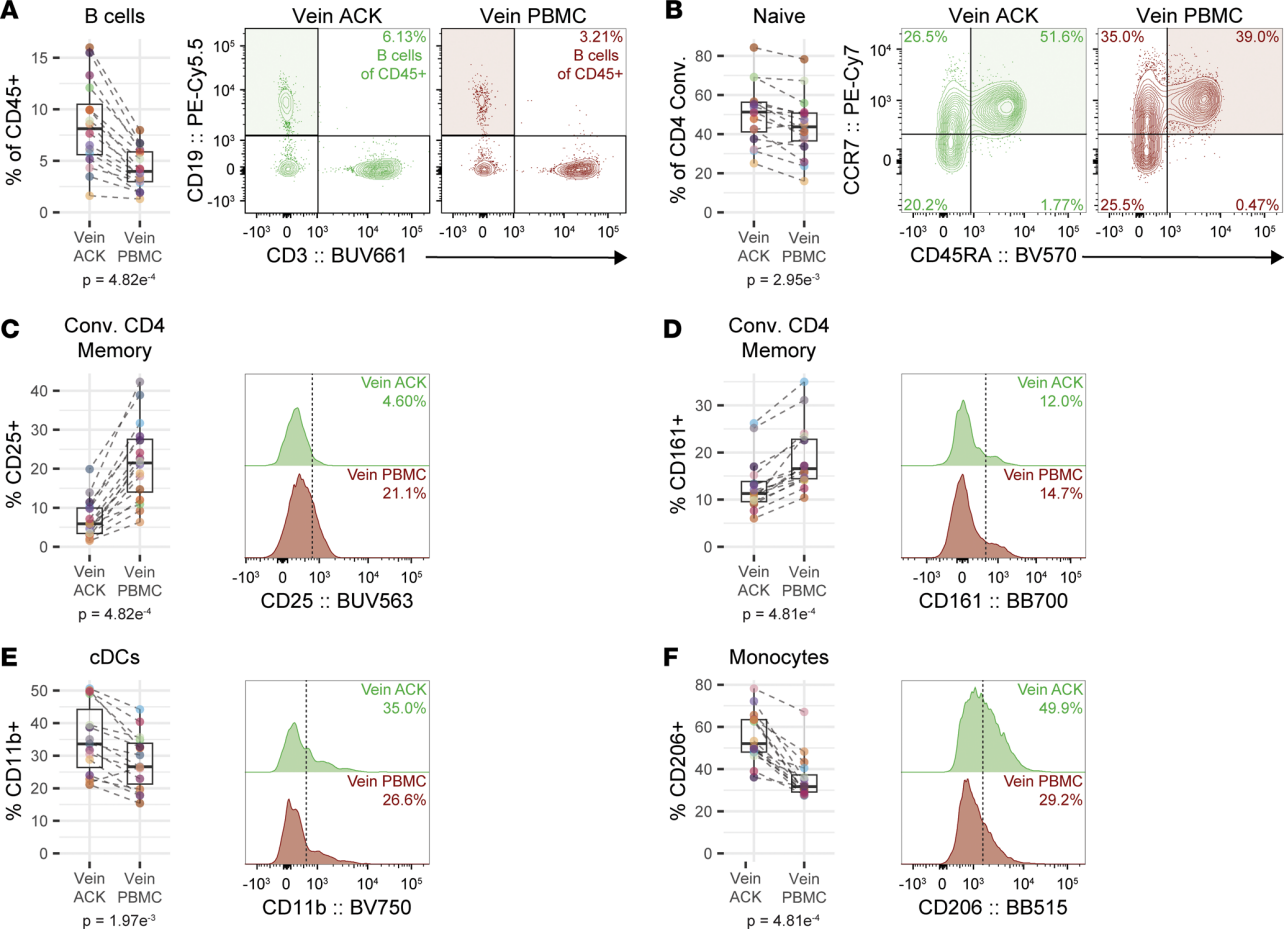
Composition and phenotype of immune cells vary with different blood-processing protocols. (**A**–**F**) Blood obtained from the same venipuncture blood draw was split and processed by both ACK lysis to produce a whole sample (Vein ACK) and by Ficoll density gradient to produce a PBMC sample (Vein PBMC). (**A**) Quantification and representative plot of B cells as a frequency of CD45^+^ non-neutrophils (*n* = 16). (**B**) Quantification and representative plot of naive conventional CD4^+^ T cells as a frequency of conventional CD4^+^ T cells (*n* = 16). (**C**–**F**) High-quality samples were used for phenotyping analysis: samples with greater than 70% viability and subsets where there were 20 or more cells. Memory conventional CD4^+^ T cells (*n* = 16), cDCs (*n* = 15), and monocytes (*n* = 16). (**C** and **D**) Quantification and representative plot of CD25 (**C**) and CD161 (**D**) on memory conventional CD4^+^ T cells. (**E**) Quantification and representative plot of CD11b on cDCs. (**F**) Quantification and representative plot of CD206 on monocytes. Data shown are from 2 independent experiments. Statistical analyses were performed using Wilcoxon’s signed-rank test.

We next asked how comparable ACK lysis, which is routinely used to lyse RBCs in mouse model experiments, is with Ficoll gradient processing aside from the depletion of polymorphonuclear cells. Blood obtained by venipuncture was split into 2 aliquots and processed via Ficoll gradient or ACK lysis. Both samples were then analyzed using our APC and T cell flow cytometry panels. Granulocytes, specifically neutrophils and eosinophils, are depleted when generating PBMCs (27). To correct for potential neutrophil contamination in our ACK-lysed samples, CD16^hi^ and CD45^lo^ cells were removed from the analysis (Supplemental Figure 1A). Despite this correction, we observed changes in the major immune cell composition when comparing ACK-lysed samples to PBMC samples, where B cell frequencies (as a percentage of CD45^+^ non-neutrophil cells) were doubled in ACK-lysed samples (Figure 2A). Furthermore, for conventional CD4^+^ T cells, we observed a slight increase in the relative proportion of naive CD4^+^ T cells (percentage of total conventional CD4^+^ T cells) between the ACK-lysed and PBMC samples (Figure 2B). In addition to relative immune cell frequencies, we observed differences in staining for phenotyping markers. For memory conventional CD4^+^ T cells, we found that the frequency of CD161^+^ and CD25^+^ cells was higher in PBMC samples (Figure 2, C and D). The frequency of CD11b^+^ cDCs and CD206^+^ monocytes was increased in ACK compared with PBMC samples (Figure 2, E and F). Collectively, these data illustrate that the method by which blood is processed introduces bias that appears at both the subset and phenotype level in immune cells when interrogated by flow cytometry. These data highlight the need for highly standardized blood processing in studies to ensure that processing-related changes are not interpreted as biological changes.

### A Tasso+ drawn blood sample yields a sufficient number of cells for subsetting even after shipping.

**Figure 3:**
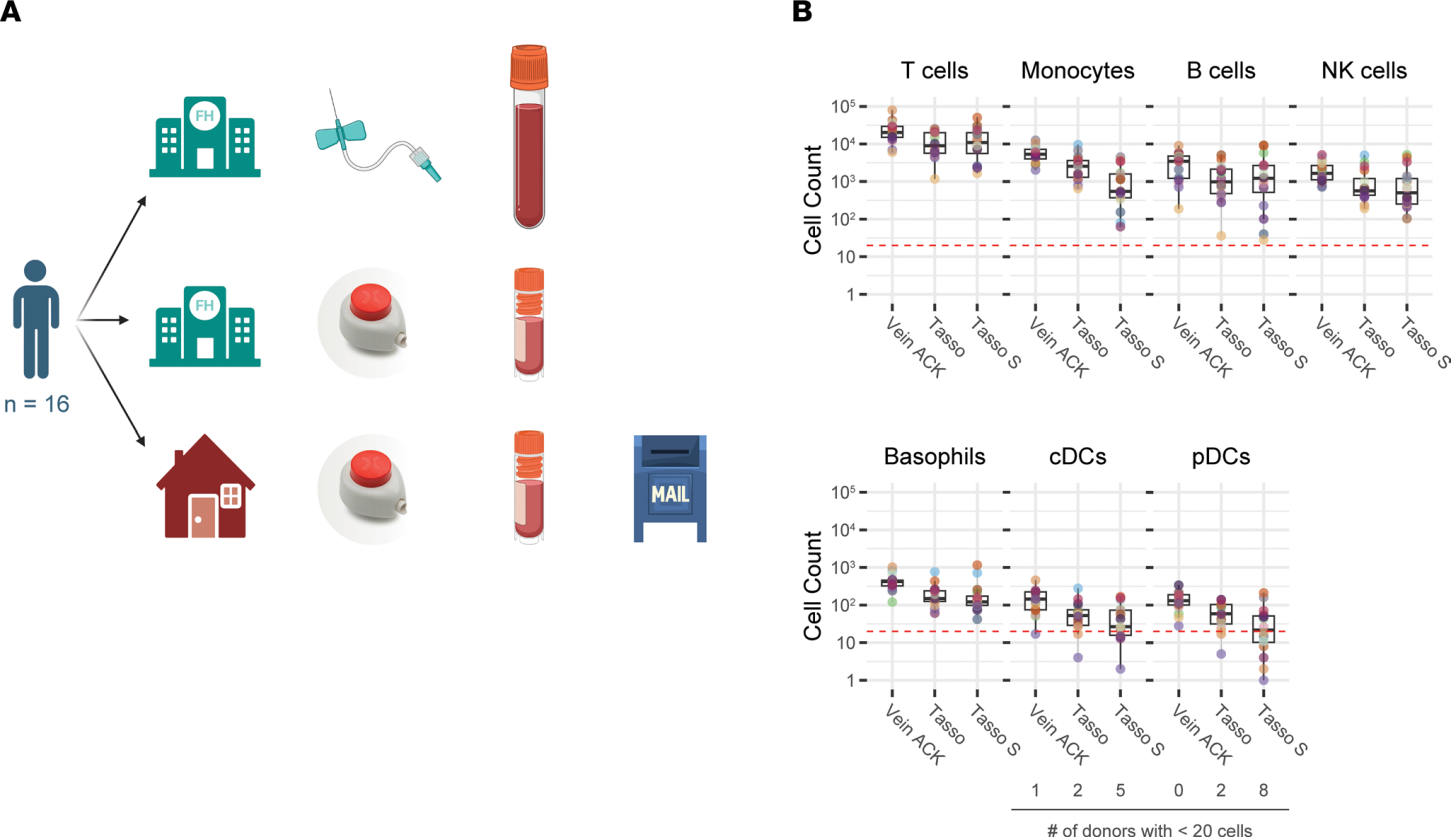
A Tasso+ blood sample yields a sufficient number of cells for subsetting even after shipping. (**A**) Overview of experimental design. (**B**) Cell counts of major immune cell subsets from venipuncture-collected ACK-lysed whole blood (Vein ACK, 0.5 mL); ACK-lysed whole blood collected with Tasso+ at the center and processed immediately (Tasso, 1/2 collected volume per panel, up to 0.5 mL per panel); and ACK-lysed whole blood drawn at home with Tasso+ and shipped to the center (Tasso S, 1/2 collected volume per panel, up to 0.5 mL per panel). *n* = 16. Data shown are from 2 independent experiments. Dotted threshold placed at 20 cells: the cutoff used for including donors for further immune cell phenotyping.

To test the feasibility of implementing the Tasso+ device in decentralized studies that utilize flow cytometry, participants drew a blood sample with the Tasso+ device in our study clinic under supervision, and then received a second Tasso+ device to take home, collect their own blood without supervision, and then mail back to the clinic via the United States Postal Service (USPS) in a padded envelope. Ice packs or other methods to control the temperature were forgone to minimize cost and keep the workload for participants as minimal as possible (Figure 3A). The Tasso+ collection tubes were coated with a dry EDTA anticoagulant provided by the manufacturer, but no stabilization buffers were added. This approach ensured that there was no possibility of inadvertently exposing participants to buffer via the punctured skin. For each donor, we were able to recover all major immune cell subsets of the peripheral blood in the Tasso+ device sample taken in the clinic: T cells, monocytes, B cells, NK cells, basophils, cDCs, and pDCs. With respect to the 16 Tasso+ samples obtained in the clinic, 2 donors’ cDCs and 2 donors’ pDCs were below our cell number cutoff (≥20 cells) to perform further phenotyping (Figure 3B). These data highlight that even assessment of rare cell populations such as cDCs and pDCs can be feasible for most if not all Tasso+ samples (as highlighted earlier, all of our Tasso+ samples were split in half for analysis with 2 flow cytometry panels), and that abundant populations such as T cells, B cells, NK cells, and monocytes can be reliably interrogated using blood collected with a Tasso+ device. In the shipped Tasso+ samples, we observed a 50% or more decrease in the recovery of monocytes, cDCs, and pDCs when compared with the Tasso+ samples acquired in the clinic. Conversely, T cells, B cells, and NK cells maintained cell counts when shipped. Although present at low cell numbers and derived from the myeloid linage, basophils also showed comparable cell counts in the shipped Tasso+ sample. Therefore, these data suggest by recovery and robust cell numbers that T cells, B cells, and NK cells may be candidate cell subsets that are suitable for remote immune monitoring.

### T cell viability and subset distribution remain stable over shipment at ambient temperatures.

**Figure 4:**
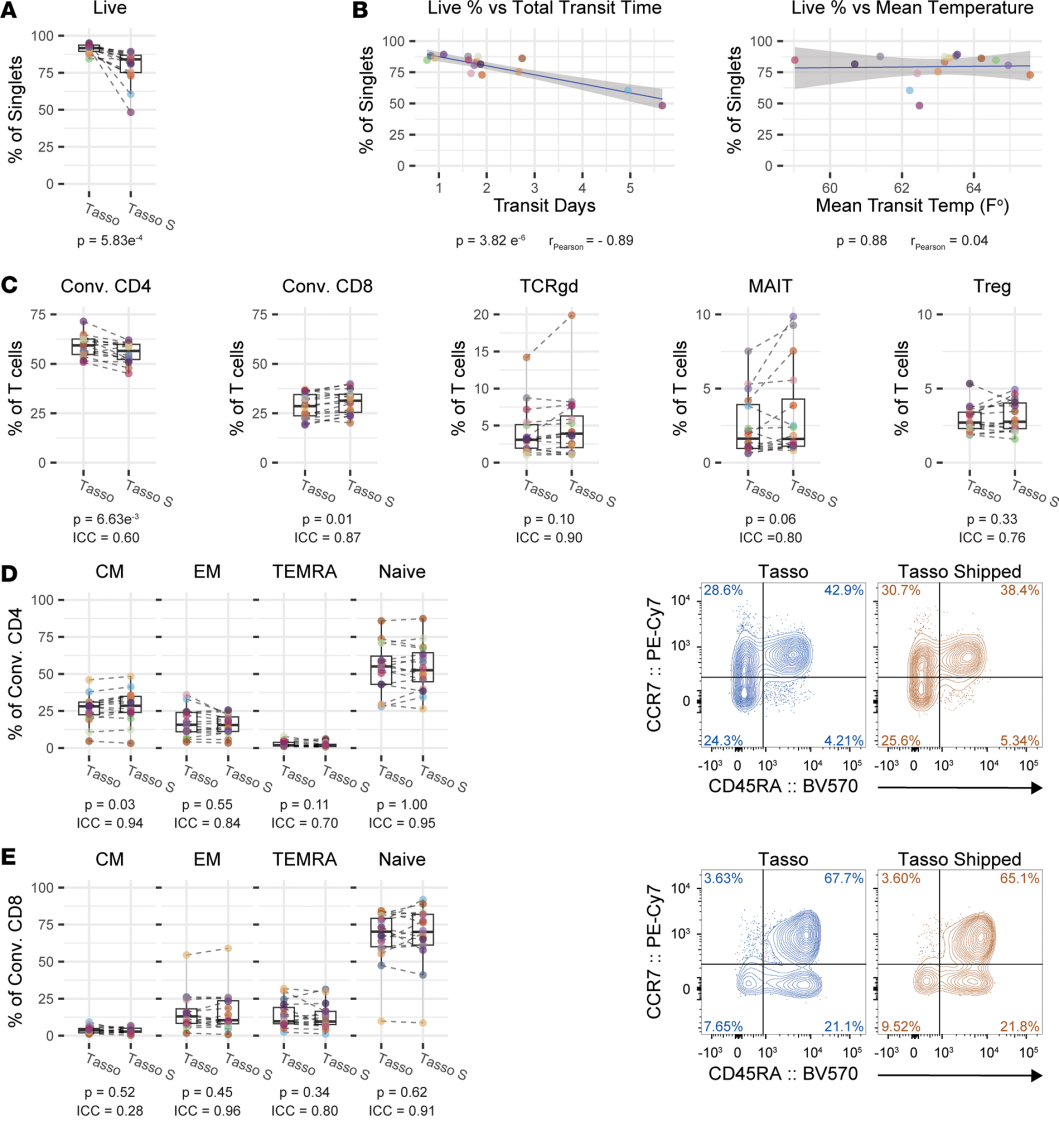
T cell viability and subset distribution remain stable after shipment at ambient temperatures. (**A**) Quantification of viability comparing ACK-lysed whole blood drawn at the center with Tasso+ and processed immediately (Tasso) and ACK-lysed whole blood drawn at home with Tasso+ and shipped to the center (Tasso S). *n* = 16. (**B**) Linear regressions testing the relationship of viability to the length of time in transit during shipment or mean temperature experienced during shipment for Tasso S samples (*n* = 16). (**C**) Quantification of T cell subsets as a frequency of total T cells (*n* = 16). (**D** and **E**) Quantification and representative plots of memory subsets for conventional CD4^+^ T cells (**D**) and conventional CD8^+^ T cells (**E**) (*n* = 16). Data shown are from 2 independent experiments. Statistical analyses were performed using Wilcoxon’s signed-rank test, intraclass correlation coefficient, and Pearson’s correlation test.

We next wanted to assess the impact of transit time on cell viability. The majority of the shipped samples were received within 48 hours (median 42.42 hours, range 18.03–135.98 hours) from the time the Tasso+ device was used at home. By retrieving weather information from National Oceanic and Atmospheric Administration (NOAA) stations in the regions the samples traveled through, we were able to determine the average ambient temperature each sample was exposed to during the time of its transit (Supplemental Table 2). As would be expected, samples collected at home with the Tasso+ device and mailed through USPS had a slight decrease in the median viability of gated lymphocytes at 84.05% with increased variability (IQR 11.6%) compared with samples collected in the clinic with Tasso+ and immediately processed (91.65%, IQR 4.225) (Figure 4A). Time in transit showed a significant negative correlation with viability, whereas the average temperature experienced showed no correlation (Figure 4B). It is noteworthy that the ambient temperatures around Seattle, Washington, the location of the clinic and proximal to donors’ home addresses, at that time were similar to room temperature, which we further elaborate on in the Discussion.

We next assessed whether shipping Tasso+ samples would skew the relative abundances of cell populations. We observed a significant decrease in the frequency of monocytes and pDCs and a concurrent relative increase in the frequency of T cells and B cells (Supplemental Figure 5A). This increase was likely due to the resiliency of T cells and B cells to cell death over the shipment period, as the whole numbers of T cells and B cells had little deviation between the Tasso+ sample collected at the clinic and the Tasso+ sample collected at home and mailed in (Figure 3B). Additionally, we observed large changes in the distribution of subsets and expression of phenotyping markers within the NK cell, cDC, and monocyte compartments (Supplemental Figure 5, B and D–F). In line with the stability of T cells in terms of numbers, the T cell compartment distribution was also relatively stable between the Tasso+ samples with and without shipment. We observed a minor reduction in conventional CD4^+^ T cells and a small increase in conventional CD8^+^ T cells, while Treg cells, MAIT cells, and TCRgd T cells remained unchanged (Figure 4C). Additionally, memory subsets of conventional CD4^+^ T cells and conventional CD8^+^ T cells remained largely unchanged (Figure 4, D and E). The one statistically significant alteration was a minor increase in central memory conventional CD4^+^ T cells; regardless, this population also displayed excellent reproducibility (ICC 0.94) (Figure 4D). Memory and naive B cell frequencies were also largely unchanged with shipment (Supplemental Figure 5C). These data highlight that EDTA-coated tubes are not sufficient to maintain viability in myeloid cells when processing is delayed, whereas T cells were stable in regard to subset distribution.

### Phenotypic states of T cells are largely congruent with and without shipment.

**Figure 5:**
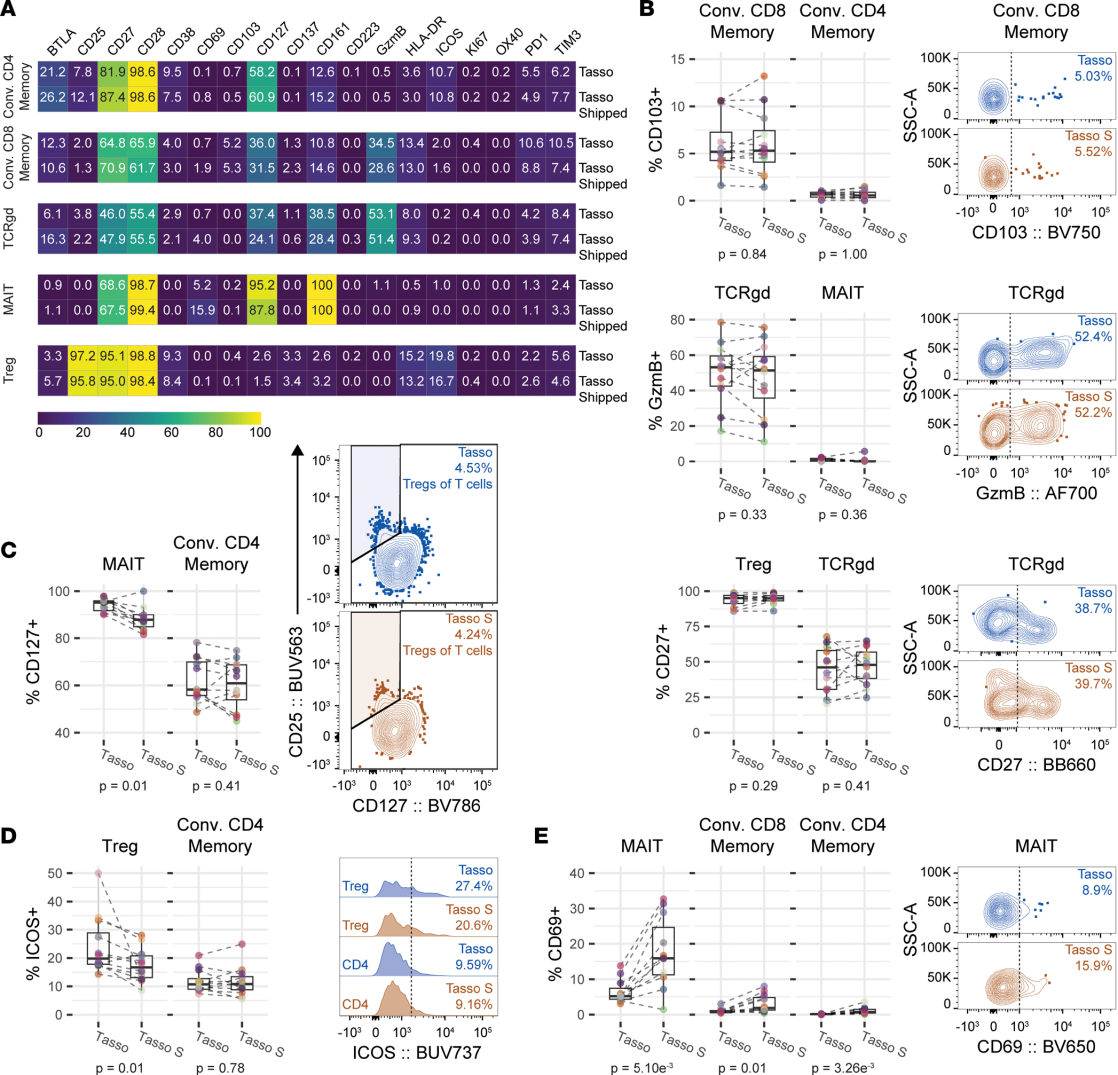
Phenotypic states of T cells are largely congruent with and without shipment. (**A**–**E**) High-quality samples were used for phenotyping analysis: samples with greater than 70% viability, samples where the transit time of the shipped Tasso+ sample was less than 48 hours, and T cell subsets where there were 20 or more cells. Memory conventional CD4^+^ T cells (*n* = 12), memory conventional CD8^+^ T cells (*n* = 12), TCRgd T cells (*n* = 12), MAIT cells (*n* = 11), and Treg cells (*n* = 12). (**A**) Heatmap reporting the medians of phenotyping markers for T cell subsets from ACK-lysed whole blood drawn at the center with Tasso+ and processed immediately (Tasso) and ACK-lysed whole blood drawn at home with Tasso+ and shipped to the center (Tasso S). (**B**) Quantification and representative plots of CD103 for memory conventional CD4^+^ T cells and memory conventional CD8^+^ T cells, Granzyme B for TCRgd T cells and MAIT cells, and CD27 for Treg cells and TCRgd T cells. (**C**) Quantification of CD127 for MAIT cells and memory conventional CD4^+^ T cells and representative plots of Treg gating utilizing CD127. (**D**) Quantification and representative plots of ICOS for Treg cells and memory conventional CD4^+^ T cells. (**E**) Quantification and representative plot of CD69 for MAIT cells, memory conventional CD8^+^ T cells, and memory conventional CD4^+^ T cells. Data shown are from 2 independent experiments. Statistical analyses were performed using Wilcoxon’s signed-rank test.

We next further assessed the phenotypic stability of T cells. We observed a large congruency in T cell phenotyping for all T cell subsets when comparing shipped and lab-processed Tasso+ blood for an individual (Figure 5A). These included CD103 as a marker of circulating tissue-resident T cells, CD27 and CD28 as markers informing further effector status and activation, and Granzyme B indicating cytotoxic potential (Figure 5B). Regulatory markers BTLA, PD-1, and TIM3 were also unchanged. CD127, the IL-7 receptor α chain, allows T cells to respond to IL-7 and is important for both their development and maintenance and is found on both memory and naive populations as well as innate and conventional T cells (28). The primary use of CD127 in our panel was to distinguish Treg cells that were CD25^+^CD127^lo^. We observed a decrease in CD127^+^ cells in the MAIT and TCRgd T cell population. However, we did not observe this reduction in memory conventional CD8^+^ T cells nor memory conventional CD4^+^ T cells (Figure 5C). Importantly, we did not observe a change in the frequency of Treg cells (Figure 4C), illustrating that reduction in CD127^+^ cells in the MAIT and TCRgd T cell populations did not affect the use of CD127 as a Treg cell lineage marker within the panel.

Detection of biomarkers indicating a T cell activation state was minimally decreased (3% decrease or less) on specific T cell subsets for the shipped Tasso+ device samples: CD38 on memory conventional CD4^+^ T cells and TCRgd T cells; HLA-DR on memory conventional CD4^+^ T cells, memory conventional CD8^+^ T cells, and Treg cells; and ICOS on Treg cells. However, memory conventional CD4^+^ T cells maintained similar expression of ICOS when shipped (Figure 5D). CD69 expression increased within certain T cell subsets when shipped. CD69 is often used to denote T cells that have undergone recent activation by either TCR engagement or in response to soluble inflammatory factors (29). The frequency of CD69^+^ cells increased substantially in MAIT cells and TCRgd T cells, but only modestly in conventional CD4^+^ T cells and conventional CD8^+^ T cells (Figure 5E). Together, these data illustrate that T cell phenotypes under the parameters we tested are minimally altered by shipment.

### Cytometry control allows for reproducible assessment over longitudinal studies.

**Figure 6:**
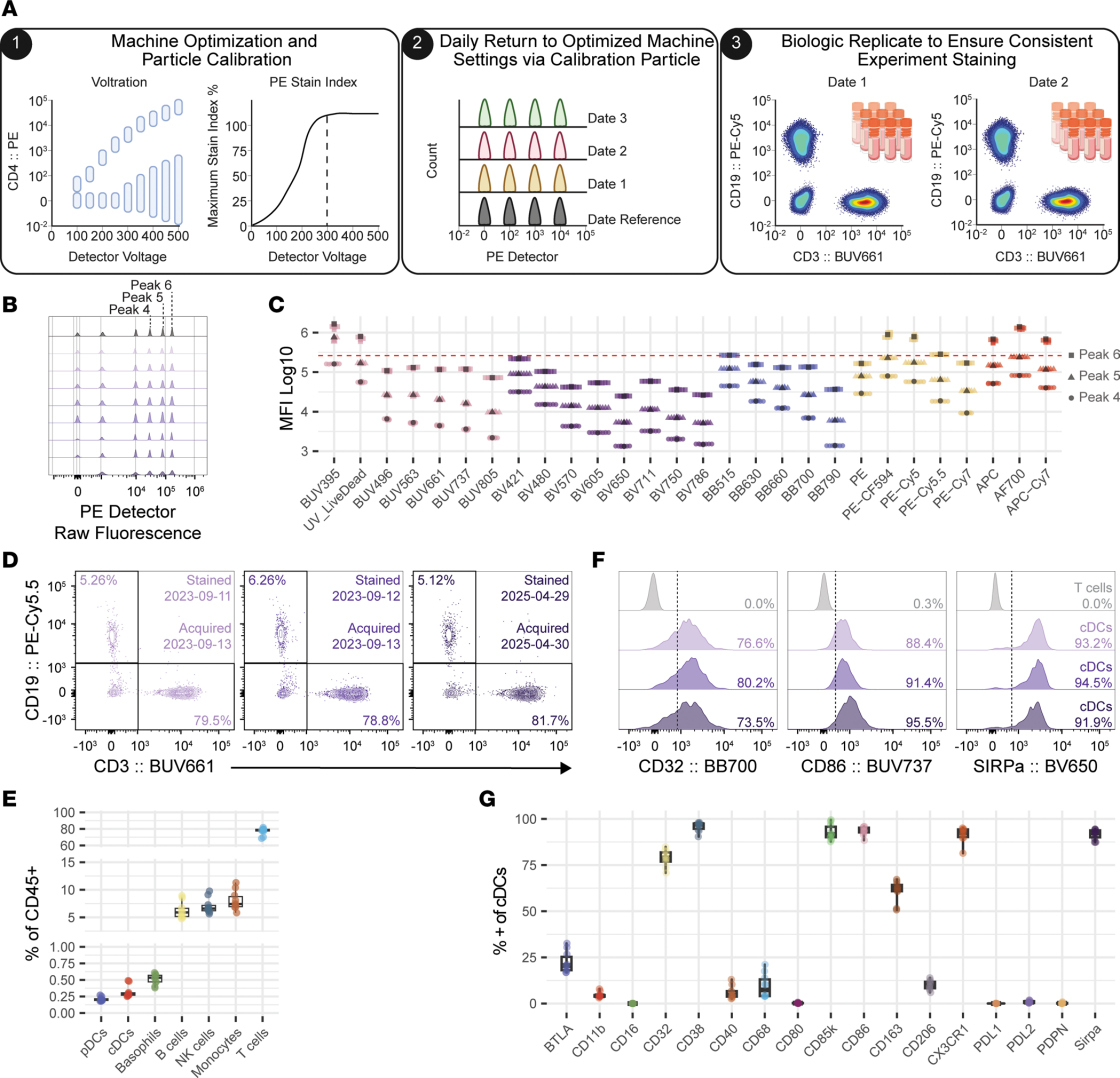
Cytometry controls allow for reproducible assessment for long-term longitudinal studies. (**A**) Overview of cytometry controls used to determine optimization and standardization of machine settings, and reagent or assay performance. (**B** and **C**) Representative plot of 6-peak Ultra Rainbow Calibration Particle raw fluorescence in the PE detector (**B**) and quantification of raw fluorescence of peaks 4, 5, and 6 across all detectors (**C**). Acquisitions took place across 2 years (reference in black and 8 subsequent acquisitions on separate days in color, *n* = 9). (**D** and **E**) Representative plot of T cell and B cell gating with frequencies of CD45^+^ non-neutrophils (**D**) and quantification of all major immune cell lineage frequencies among CD45^+^ non-neutrophils in the same biological replicate PBMC (**E**) (biological replicate stained on 10 separate days and acquired on 8 separate days, *n* = 10). (**F**) Representative plot of CD32, CD86, and SIRPα on cDCs. Color coding in **F** matches dates of staining in **D**. T cells included in **F** from September 11, 2023 with staining in gray for histograms as a negative cell population control. (**G**) Quantification of all phenotyping markers on cDCs (*n* = 10).

For many clinical studies, blood samples can be cryopreserved and later analyzed in batches to minimize technical variability that can occur between experiments. Analysis for longitudinal studies or immune monitoring may need to occur in an ongoing manner but must be able to discern biological changes from technical variation across experiments. Multiple sources of variation exist for flow cytometry that can make longitudinal studies difficult (30). Cytometry controls (Figure 6A) allow for the optimization of machine settings, daily standardization to return to optimal machine settings, and the ability to pinpoint whether reagents or staining procedures are the source of variation between batches (31). To assess the stability of our high-dimensional flow cytometry panels on an instrument over time, we analyzed an aliquot of the same biological replicate on 9 days over the span of 1.5 years. The sample was stained with our APC panel and acquired over 8 acquisition days (Supplemental Table 3). First, as has been previously described, we optimized the voltage gain for each detector by a modified voltage titration on cells (32) (example given in Supplemental Figure 6) and calibrated the optimized setting to 6-peak Ultra Rainbow Calibration Particles, which emit light across all detectors for a given cytometer (33). We found that consistent machine settings can be returned to each of the acquisition days by setting detector gains to reach a target MFI of the calibrated calibration particle (Figure 6, B and C). We also acquired multiple experiments on the same day to tease apart when variation is due to the performance of the assay or the machine settings (Figure 6D). Because assay performance is consistent, we demonstrated that standardized gates and thresholds for batches collected longitudinally can be used for both antigens that appear bimodal, such as lineage markers (Figure 6, D and E), and antigens with continuous profiles, like those seen in many phenotyping markers (Figure 6, F and G), with limited variation. Given that longitudinal or immune surveillance studies may take place across multiple acquisition days that may span years, our data demonstrate that it is technically feasible to acquire highly consistent data over long periods of time. These data also highlight the benefit of running a technical control (such as PBMCs from a leukapheresis donor) with every experiment staining procedure (31).

## Discussion

High-parameter flow cytometry is ideally suited to interrogate samples with limited cell numbers such as the low volume of capillary blood obtained using a Tasso+ device (34). We initially addressed the question of whether capillary blood is identical to venous blood using 2 high-parameter flow cytometry panels to interrogate the APC and T cell populations in depth. Although studies have described differences between capillary and venous blood (35–38), we found no substantial differences with regard to immune cell subsets and phenotypes with our flow cytometry panels, highlighting that the use of capillary blood is feasible for immunological studies. Furthermore, we addressed whether the relatively low blood volume that is collected with a Tasso+ is sufficient to assess rare populations such as cDCs, pDCs, MAIT cells, and TCRgd T cells. Importantly, our data highlight that robust identification of even a rare blood cell population is possible with the typical 100–1,000 μL of blood collected with a Tasso+ device.

Given this limited blood volume, standard blood-processing methods are not well suited due to the concern of substantial cell loss. Blood processing is a source of technical variability (39). In general, the methods used to process biological samples can affect cell recovery and phenotype. Enzyme-mediated digestion of tissues can alter a cell phenotype (40), influence which cells are released from a tissue (41), and potentially cleave cell-surface proteins required for phenotyping (42, 43). Similarly, we found that blood processed by ACK lysis or by a Ficoll density gradient to produce a PBMC sample differed in regard to composition of some cell subsets, including a 2-fold change in B cell frequencies, and staining of phenotyping markers (CD25 on memory CD4^+^ T cells, CD206 on monocytes; Figure 2, C and F). Early reports initially observed incongruencies between blood processed by only RBC lysis and Ficoll-like density gradients (44–47). Moreover, differences have also been observed when different formulations of lysis reagents were used (48). Most of these early studies could only interrogate a limited depth for immunophenotyping, observing differences related to size by light scatter parameters and major immune subsetting with few fluorescent parameters available at the time. Our work provides evidence that alterations between blood processed by ACK lysis and Ficoll-like density are observable even when interrogating more homogenous cell subsets such as conventional CD4s, cDCs, and monocytes. This also raises the question of whether there are incongruencies between human and mouse studies because human blood is routinely processed by Ficoll-like density gradients, whereas mouse blood and spleen are routinely processed by ACK lysis. Additionally, these data highlight the importance of the development of methodologies that are applicable to large and small quantities of input and may be able to isolate cells with limited side effects, such as size exclusion by microfluidics (49).

In our study design, we chose to stabilize whole blood with only a dry EDTA coating already in the collection tube and to ship via USPS at ambient temperatures to (a) prevent any additional processing or packaging requirements that the participant needed to performed, (b) assess whether the most cost-efficient shipping approach is feasible, and (c) pinpoint any potential requirements for stabilization or preservation buffers. In line with a study from Savage et al. (50) that assessed PBMC composition by flow cytometry when kept at room temperature up to 18 hours after blood draw, we found remarkable consistency in the composition of the T cell compartment between Tasso+ samples acquired at the clinic and those acquired at home and mailed in with a median of 42 hours in transit before processing. These data indicate that ABCDs are suitable to study changes in the T cell pool by flow cytometry even without stabilization buffers. Assessing biomarkers of T cell activation and exhaustion would be important for any clinical study interrogating the immune response to infections (4), therapies (51), and vaccines (52–54). Of note, delays in processing can greatly affect single-cell transcriptomics (50). Not observing a similar change on the protein level in T cells could be due to the stability of already expressed protein.

Although T cells maintained viability and phenotypic stability during the shipping process, we observed a decrease in total monocytes and pDCs during shipment, along with a change in the relative distribution of monocytes, cDCS, and NK cells. These data highlight the importance of using a stabilization buffer to preserve the integrity of the myeloid population during shipping. Reagents used to preserve qualities of peripheral blood cells can be divided into chemical fixatives used for processed cells (paraformaldehyde, glutaraldehyde, glyoxal, ethanol, methanol), and stabilizers of whole blood (Cyto-Chex and TransFix). These reagents have been reviewed and extensively tested (55). Whole-blood stabilizers would be the ideal for implementation in decentralized studies because they could be provided in the blood collection tube or mixed in with whole blood with minimal training of the participant; however, they impair staining of cell-surface proteins, disrupt initial cell subset composition, and still fail to preserve granulocytes and monocytes (56, 57). Chemical fixatives like paraformaldehyde are not usable when viable cells are required but perform better in maintaining cell subset distributions; however, some protein targets may lose the ability to be recognized by monoclonal antibodies due to loss of an epitope when proteins cross-link (58). Likewise, transcriptomic studies are not possible to date with paraformaldehyde-fixed cells due to RNA cross-linking with protein (59). New stabilizing buffers appear to be developed that allow for the interrogation of protein and RNA species while preventing the loss of myeloid-derived populations (60). Additional studies need to address whether these new buffers may be implemented in decentralized studies to regain the ability to assess myeloid populations when there are delays in blood processing. Finally, since our samples were cryopreserved after they arrived at the lab and were processed, we did not interrogate neutrophils or eosinophils, which have poor recovery upon freeze-thaw and are greatly affected by delays in processing after blood draw (55, 61).

Stabilization buffers will likely also be critically important when shipping samples in ambient temperatures that exceed room temperature. In our study, we observed no correlation between the viability of shipped Tasso+ samples and the ambient temperature that they experienced during shipment. We want to note that due to the region and to the time of year our study was performed, there was a narrow range in temperatures that our cohort of shipped Tasso+ samples (50°F–77°F) were exposed to, which did not exceed greatly what is considered room temperature (68°F–72°F). Temperature control packaging may be a solution to limit variation in the temperatures that shipped Tasso+ samples are exposed to, but this would increase the workload for participants and the per-sample cost of studies. Ideally, newly created stabilizing buffers would have performance over a range of temperatures to address preservation of blood samples across all climates.

A potential roadblock for utilizing flow cytometry for longitudinal studies is that multiple sources of variation can be present between different days of acquisition for flow cytometry analysis, which could interfere with discerning biological from technical changes. The use of cryopreserved samples allows for distributing samples evenly to control for potential batch variation. However, in longitudinal studies or immune monitoring studies, samples may need to be analyzed across different acquisition days. Importantly, we found that robust and highly reproducible longitudinal high-parameter flow cytometry is feasible when best-practice controls are in place for machine calibration, machine setup, and longitudinal batch observation.

ABCDs provide participants with the convenience of collecting their blood at home and may even be preferred over traditional venipuncture (16, 19, 62). Overall, our data provide evidence that the Tasso+ device is suitable for use with high-parameter flow cytometry to detect even rare immune cell subsets. Based on our data, we argue that the development of a stabilization buffer that preserves myeloid cells and protects cells in extreme temperatures is the last hurdle to routinely use ABCDs for remote immune monitoring.

## Methods

### Sex as a biological variable.

Sex as a biological variable was not tested because the number of enrolled participants did not allow for adequate power of the current study. The impact of sex as a biological variable in data variability or bias was controlled for by performing only paired testing, where cohort sex composition was equivalent for both testing groups.

### Isolation of leukocytes from peripheral blood and ACK lysis of whole blood.

Whole blood was collected by venipuncture into a Vacutainer with ACD Solution A (Becton Dickinson, 364606) and from capillary beds using the Tasso+ ABCD into a tube containing a dried coating of EDTA. Blood collected from venipuncture was processed by 2 methods: generation of PBMCs by density gradient and by enrichment of WBCs by RBC lysis. Blood collected using the ABCD was only processed by enrichment of WBCs by RBC lysis.

For generation of PBMCs, 1 mL of whole blood was diluted with DPBS (Gibco, 14190-144) and then slowly added to a 15 mL SepMate tube (STEMCELL Technologies, 85414) containing 4.5 mL of Lymphoprep (STEMCELL Technologies, 07851). The SepMate tube was then centrifuged at 1,200*g* for 16 minutes with brake set to a medium setting (5 of 9). The buffy coat was decanted into a new 15 mL tube and DPBS was added to reach a total volume of 10 mL. The sample was centrifuged at 400*g* for 5 minutes with the brake fully on (9 of 9). The sample was decanted, resuspended in 10 mL of DPBS, and counted using trypan blue exclusion (Gibco, 15250-061) on a Bio-Rad TC20 automated cell counter with a size range of 6–12 μm. The sample was centrifuged at 400*g* for 5 minutes with the brake fully on. The sample was decanted, resuspended in 1 mL of Recovery Cell Culture Freezing medium (Gibco, 12648-010), transferred to a cryovial, placed in a StrataCooler at –80 C for 1 day, and then transferred to liquid nitrogen for long-term storage until use.

For generation of enriched WBCs, 1 mL of blood collected by venipuncture or the entire contents collected by ABCD were diluted with 11 mL of ACK lysis buffer (Gibco, A10492-01) in a new 15 mL tube and incubated at room temperature for 3–5 minutes. The sample was centrifuged at 400*g* for 5 minutes with the brake fully on. The sample was decanted, resuspended in 10 mL of DPBS, and counted using trypan blue exclusion on a Bio-Rad TC20 automated cell counter with a size range of 6–12 μm. The sample was centrifuged at 400*g* for 5 minutes with the brake fully on. The sample was decanted, resuspended in 1 mL of Cell Recovery Freezing medium, transferred to a cryovial, placed in a StrataCooler at –80 C for 1 day, and then transferred to liquid nitrogen for long-term storage until use.

### Flow cytometry.

Flow cytometry panel design and execution were conducted using best practices. All staining was performed at room temperature in round-bottom 96-well plates. After thawing, cells were incubated with Trustain FcX (BioLegend, 422302), a blocking reagent for Fc receptors, and Live/Dead Fixable Blue Dead Cell stain (Invitrogen, L23105) in DPBS for 15 minutes. Cells were then washed once; 150–200 μL of PBS supplemented with 2% FBS (hereinafter referred to as staining buffer) was added to cells, centrifuged at 400*g* for 5 minutes, and then the supernatant decanted. To prevent known steric hindrance interactions between antibody labeling of cell-surface markers, sequential staining was performed to preference the resolution of certain markers. In the T cell panel, this was done by staining the MR1 tetramer (ligand: 5-OP-RU) and γδ TCRs before other surface markers, and in the APC panel, staining CX3CR1 before all other surface markers. All antibodies were titrated and used at optimal concentration for resolution in respective panels. Supplemental Table 1 displays the full list of antibodies used, the panel and mix they belong to, and the titers at which they were used. After live/dead staining and blocking of Fc receptors, cells were incubated with 50 μL of surface antibody master mix 1, and then incubated for 30 minutes in staining buffer supplemented with Brilliant Stain Buffer Plus (BD Biosciences, 566385) to prevent polymer dye aggregates. Cells were then washed once; 150–200 μL of staining buffer was added to cells, centrifuged at 400*g* for 5 minutes, and then the supernatant decanted. Cells were then incubated with 50 μL of surface antibody master mix 2, and then incubated for 30 minutes in staining buffer supplemented with Brilliant Stain Buffer Plus. Cells were then washed twice; 150–200 μL of staining buffer was added to cells, centrifuged at 400*g* for 5 minutes, and then the supernatant decanted. For intracellular/intranuclear staining of KI67, Granzyme B, and CD68, cells were fixed with fixation/permeabilization solution (BD Biosciences, 51-2090KZ) for 20 minutes. Cells were then washed once; 150–200 μL of diluted Perm/Wash buffer (BD Biosciences, 51-2091KZ) was added to cells, centrifuged at 800*g* for 5 minutes, and then the supernatant decanted. Cells were then incubated with 50 μL of intercellular antibody master mix 1, and incubated for 30 minutes in BD Biosciences Perm/Wash buffer. Cells were then washed twice; 150–200 μL of diluted BD Biosciences Perm/Wash buffer was added to cells, centrifuged at 800*g* for 5 minutes, and then the supernatant decanted. Cells were finally resuspended in staining buffer and stored at 4°C until acquisition.

Single-stain controls were prepared for each experiment and treated experimentally the same as the full stained samples. Single-stain controls were made using anti-Ig beads to bind fluorochrome-conjugated antibodies (CompBead Anti-Mouse Ig, κ, BD Biosciences, 552843; CompBead Anti-Rat and Anti-Hamster Ig κ, BD Biosciences, 552845; CompBead Plus Anti-Mouse Ig, κ, BD Biosciences, 560497), and amine-reactive beads (ArC Amine Reactive Compensation Bead, Invitrogen, A10346) to bind live/dead dyes. For each experiment, a PBMC sample from the same Leukopak donor was stained and acquired to act as a longitudinal control between batches (data not shown). For each donor, all experimental conditions (i.e., venipuncture blood collected at center, ABCD blood collected at center, ABCD blood collected at home and shipped) were collected in the same experiment to capture any variation in experimental conduct equally among all experimental groups.

All samples were acquired on a single FACSymphony A5 (BD Biosciences). The cytometer contains 30 fluorescence detectors and 5 lasers: 355 nm (65 mW), 406 nm (200 mW), 488 nm (200 mW), 552 nm (150 mW), and 628 nm (200 mW). The cytometer uses FACSDiva software. Detector gains were optimized using a modified voltration approach and brought back to standardization day to day by achieving target MFIs of 6-peak Sphero Ultra Rainbow Calibration Particles (Spherotech, URCP-38-2K).

Experiment files were exported as FCS3.1 format from FACSDiva. FlowJo (version 10.10.0) was used as a manual gating manager to determine frequencies of cellular populations and the percentage positivity of phenotyping markers. Phenotyping gate thresholds were kept constant between all experiments and all cell types.

### Sourcing of climate data to obtain ambient temperature during shipments.

The ambient temperature that devices would have been exposed to was approximated by querying climate data of a station within 10 miles of the Fred Hutchinson Cancer Center. Data were obtained from the National Centers for Environmental Information, NOAA (https://www.ncei.noaa.gov). Hourly readings from September 1, 2022 to October 8, 2022 were acquired from the Seattle Boeing Field, Washington station (Network ID WBAN24234) and time matched to the period between when each device was used and before it was received at the center.

### Statistics.

All statistics and graphical representations of tabular data were performed in R. Nonparametric measures of centeredness and variability (median, IQR, or range) are only reported and tested due to the skewedness of human datasets. A *P* value ≤ 0.05 was considered statistically significant. To assess potential differences due to multiple hypothesis corrections, a conservative testing approach was employed by performing only paired 2-group testing using a Wilcoxon’s signed-rank test. Linear regressions were tested using Pearson’s correlation coefficient test. The following libraries were used to read and organize data: *tidyr*, *dyplyr*, *readr*, and *stringr*. R base and *stats* were used for computing medians, quartiles, IQR, and range. *ggstatplot* was used for both computing statistics for testing (Wilcoxon’s signed-rank test and Pearson’s correlation test) and for graphical representation. The *irr* package was used to compute the ICCs using the *icc* function with the following parameters: model = “twoway,” type = “agreement,” unit = “single,” conf.level = 0.95. *purrr* was used to map functions over multiple variable inputs. Heatmaps were rendered using *pheatmap*. To provide additional functionality in plotting components, *viridis*, *RcolorBrewer*, and *ggplot2* were used. Graphs were formatted for publication using Adobe Illustrator.

### Study approval.

We enrolled 20 individuals to demonstrate the feasibility of proteomics analysis of live cells by flow cytometry using an ABCD shipped to research centers. The first 4 donors’ samples were used for optimization of procedures (data not shown) and the later 16 samples were used for demonstration of the procedure (Supplemental Table 2). The study was approved by the IRB of Fred Hutchinson Cancer Center, Seattle, Washington.

### Data availability.

All tabular data represented as points in graphs or summary statistics are present in the Supporting Data Values file. Flow cytometry raw data as FCS files have been deposited in ImmPort (https://www.immport.org) under study accession SDY3463. R code for generating plots and statistics has been deposited on GitHub (https://github.com/akonecny/tasso_abcd_statistics).

## Author contributions

AJK designed the research study, conducted experiments, acquired data, analyzed data, wrote the manuscript, and reviewed and edited the manuscript. FYL, AW, and MB designed the research study and reviewed and edited the manuscript. EDV conducted experiments, acquired data, and reviewed and edited the manuscript. EL, RLB, and LEK recruited participants. MP designed the research study, analyzed data, wrote the manuscript, reviewed and edited the manuscript, acquired funding, and supervised the study.

## Funding support

This work is the result of NIH funding, in whole or in part, and is subject to the NIH Public Access Policy. Through acceptance of this federal funding, the NIH has been given a right to make the work publicly available in PubMed Central.

- NIH grants R01AI123323, R01AI179712, and 1R56DE032009 (to MP).
- NIH grant P30CA015704 (to the Flow Cytometry Shared Resource [RRID: SCR_022613] of the Fred Hutchison/University of Washington/Seattle Children’s Cancer Consortium).

## Supplementary Material

Supplemental data

Supporting data values

## Version 1 04/08/2026

Electronic publication
