# Supplementary material for: An at-home blood collection device for remote immune monitoring by high-parameter flow cytometry
Source: JCI Insight. 2026 Apr 8;11(7):e201116. doi: 10.1172/jci.insight.201116 (PMC13134726; doi:10.1172/jci.insight.201116)
Supplement: Supplemental data [file jciinsight-11-201116-s172.pdf]

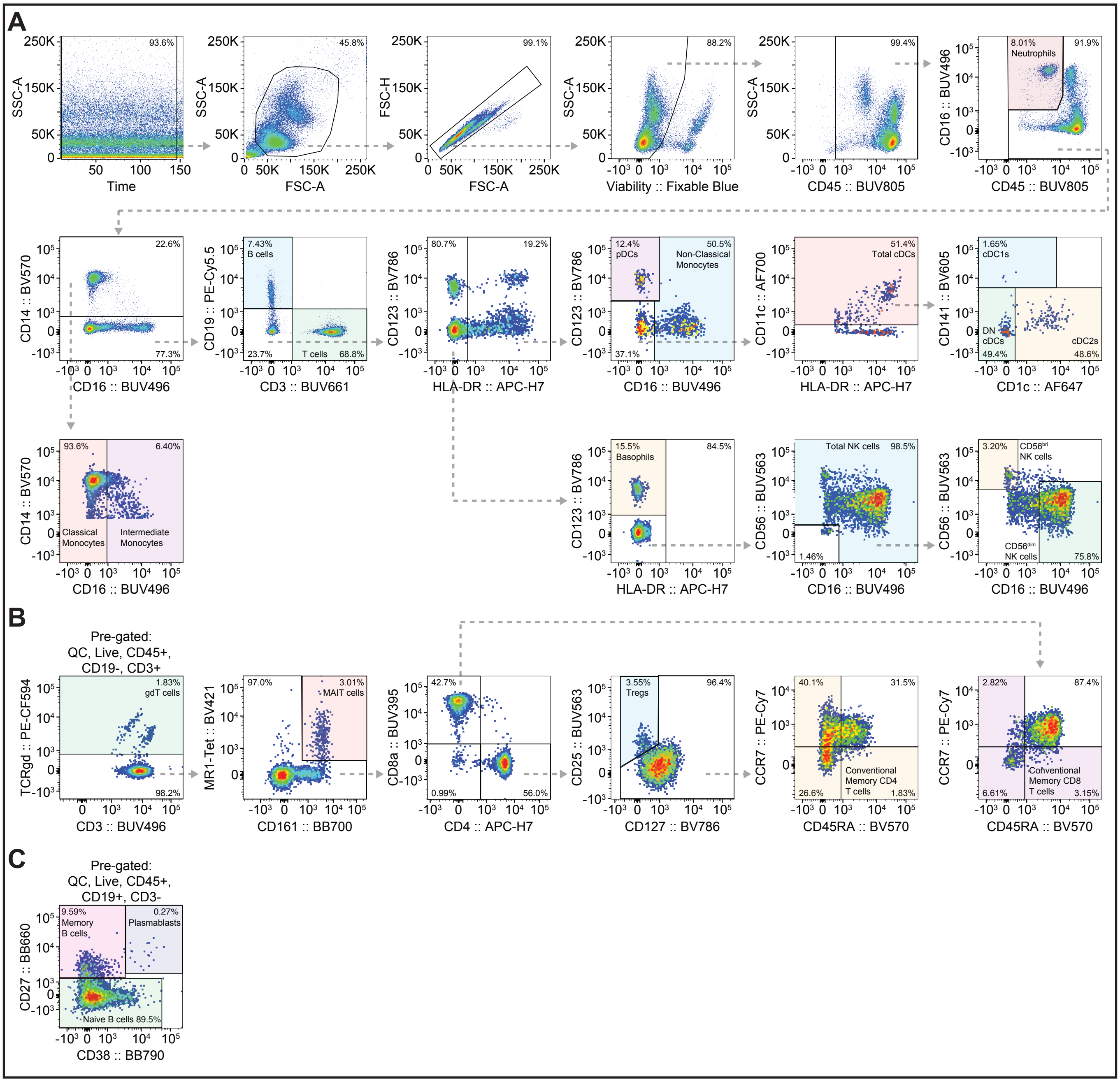

Supplementary Figure 1: ***Gating strategy for APC and T cell lineages*.**

A. Representative plots for lineage gating of cell subsets in the APC panel from a venipuncture ACK lysed whole blood sample. **B**. Representative plots for T cell subsets in the T cell panel from a venipuncture ACK lysed whole blood sample. **C**. Representative plots for B cell subsets in the T cell panel from a venipuncture ACK lysed whole blood sample.

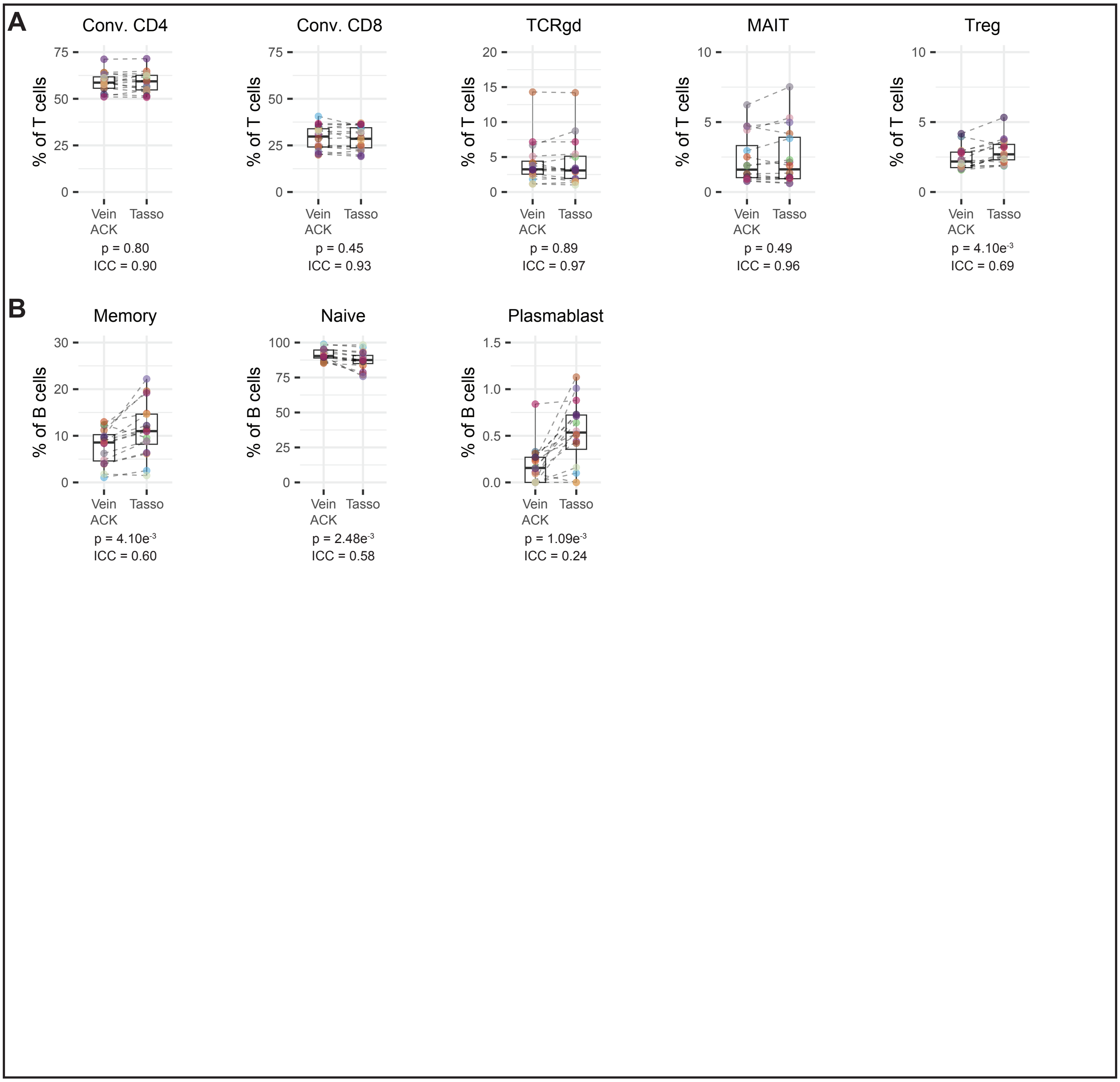

Supplementary Figure 2: ***T cells subset distributions in capillary blood and venipuncture are equivalent*.**

A. Quantification of T cell subsets as a frequency of total T cells comparing venipuncture collected ACK lysed whole blood (Vein ACK) and Tasso+ collected ACK lysed whole blood (Tasso) (n=16). **B**. Quantification of B cell subsets as a frequency of total B cells comparing venipuncture collected ACK lysed whole blood (Vein ACK) and Tasso+ collected ACK lysed whole blood (Tasso) (n=16). Data shown are from 2 independent experiments. Statistical analyses were performed using Wilcoxon signed-rank test and Intraclass Correlation Coefficient.

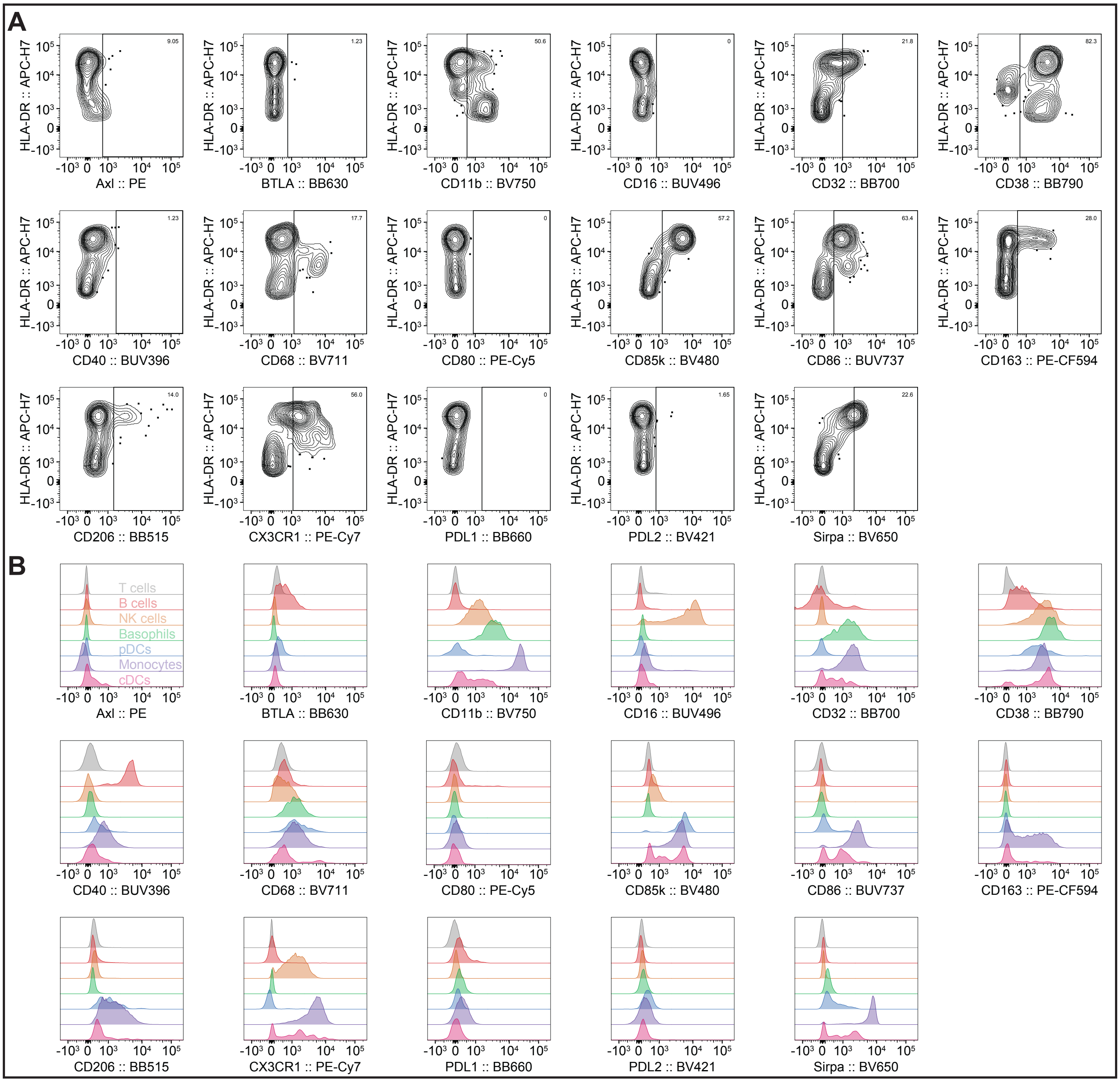

Supplementary Figure 3: ***Gating strategy for APC panel phenotyping*****.**

A. Example staining of all phenotyping markers in the APC panel on cDCs from venipuncture collected ACK lysed whole blood. The same positive gate thresholds were used for all cell subsets and all biological samples. **B**. Example staining for all phenotyping markers for all cell subsets in the APC panel; T cells (grey), B cells (red), NK cells (orange), basophils (green), pDCs (blue), monocytes (purple), and cDCs (pink).

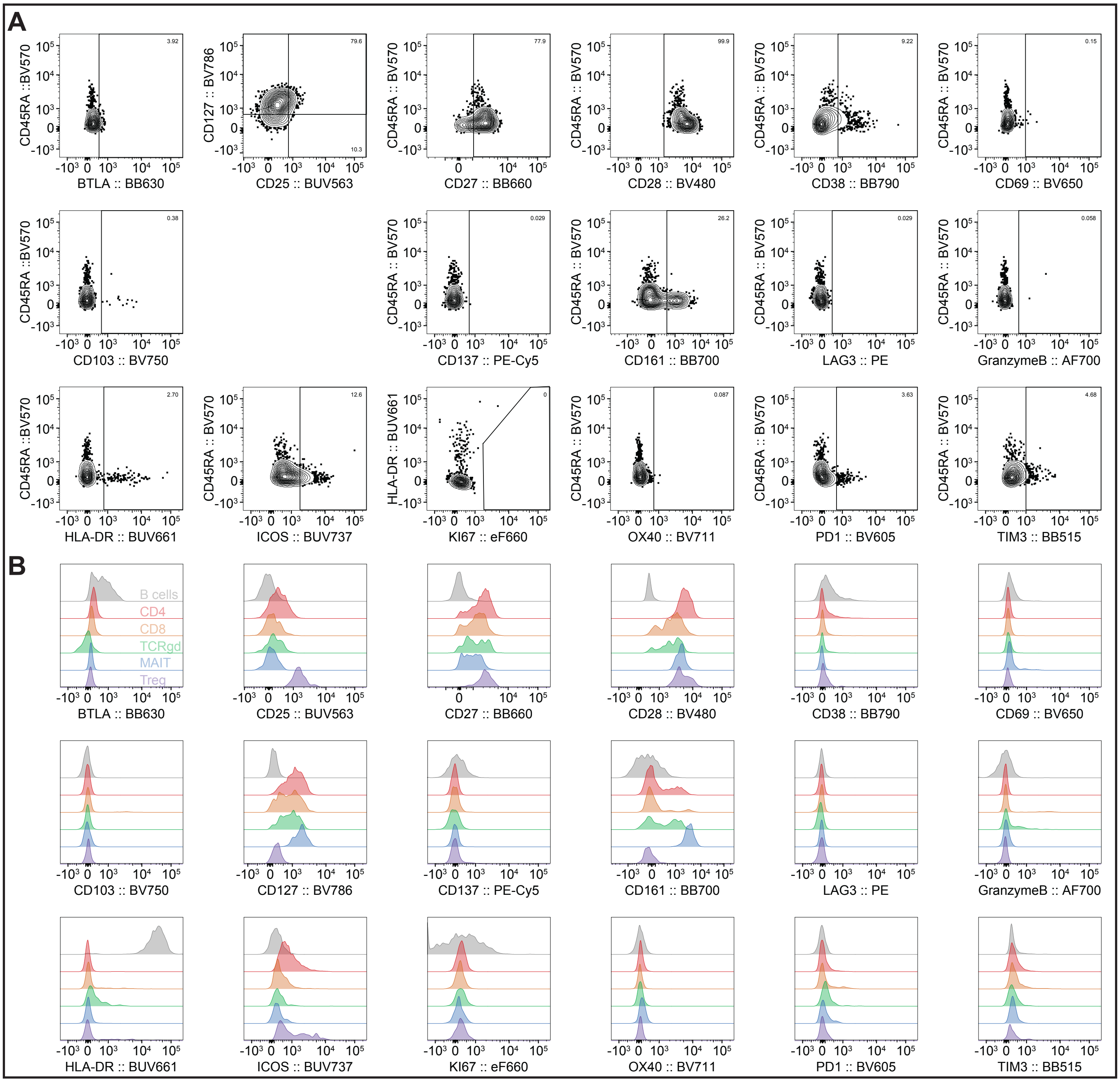

Supplementary Figure 4: *Gating strategy for T cell panel phenotyping*.

A. Example staining of all phenotyping markers in the T cell panel on memory conventional CD4 T cells from venipuncture collected ACK lysed whole blood. The same positive gate thresholds were used for all cell subsets and all biological samples. **B**. Example staining for all phenotyping markers for all cell subsets in the T cell panel; B cells (grey), memory conventional CD4 T cells (red), memory conventional CD8 T cells (orange), TCRgd T cells (green), MAIT cells (blue), and Treg cells (purple).

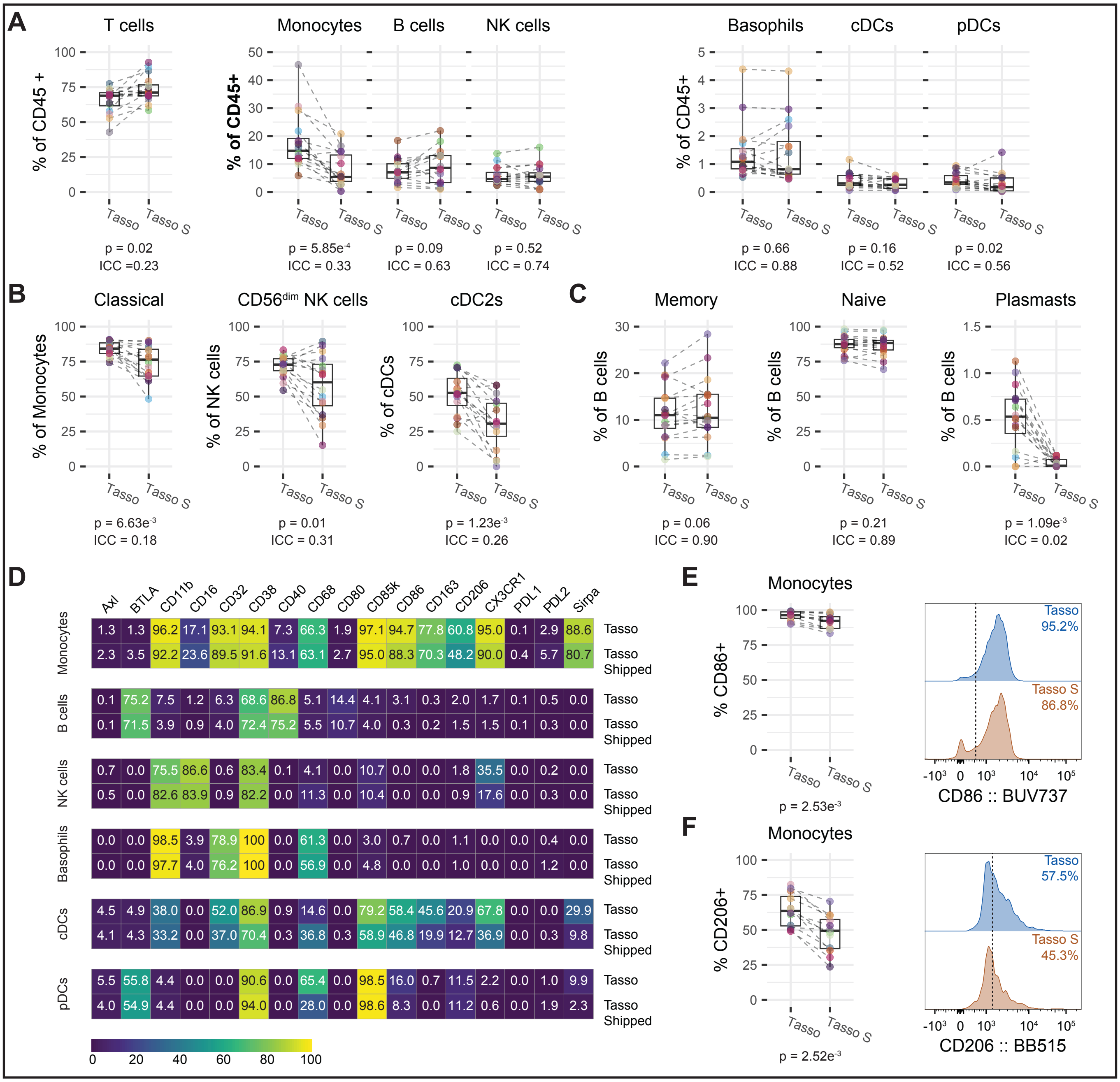

Supplementary Figure 5: ***Myeloid cell numbers prevent accurate phenotyping with low blood volume.***

A. Quantification of major immune cell subsets frequencies out of CD45+, non-neutrophils comparing Tasso+ collected ACK lysed whole blood drawn at the center and processed immediately (Tasso) and Tasso+ collected ACK lysed whole blood drawn at home and shipped to the center (Tasso S) (n=16). **B**. Quantification of classical monocytes as a frequency of monocytes, CD56dim NK cells as a frequency of NK cells, and cDC2s as a frequency of cDCs (n=16). **C**. Quantification of memory, naive, and plasmablasts as a frequency of total B cells (n=16). **D-F**. High quality samples were used for phenotyping analysis: samples with greater than 70% viability, samples where the transit time of the shipped Tasso+ sample was less than 48 hours, and subsets where there were 20 or more cells. Monocytes (n=12), B cells (n=12), NK cells (n=12), basophils(n=12), cDCs (n=8), and pDCs (n=7). **D**. Heatmap reporting the medians of phenotyping markers for cells defined by the APC panel comparing Tasso and Tasso S samples. **E&F**. Quantification and representative plots of CD86 (**E**) and CD206 (**F**) for monocytes (n=12). Data shown are from 2 independent experiments. Statistical analyses were performed using Wilcoxon signed-rank test and Intraclass Correlation Coefficient.

A

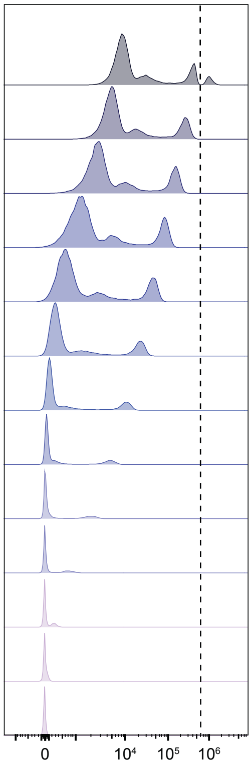

Volt	Stain Index
900	67.0
850	75.6
800	74.9
750	73.3
**700**	**74.9**
650	69.4
600	65.2
550	51.6
500	32.3
450	13.4
400	4.46
350	0.027
300	0

B

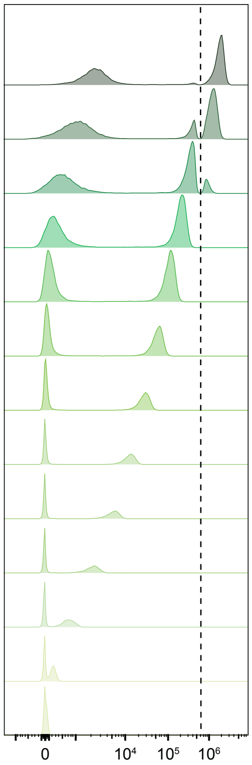

Volt	Stain Index
900	146
850	237
800	364
**750**	**392**
**700**	**377**
650	342
600	290
550	202
500	102
450	44.6
400	15.3
350	8.09
300	2.11

Supplementary Figure 6: ***Example of a voltage titration*.**

A&B. Histogram of uncompensated fluorescence of lymphocyte gated PBMC single stain CD4 on BUV563 (**A**) and PE (**B**) collected over voltage gains from 300 to 900 at 50-volt increments. Dotted line indicates where detector is off scale. Green text indicates an opitmal voltage setting in regards to stain index.

Supplementary Table 1
Flow cytometry panels used

Panel 1: T cell Panel

	Laser	Bandpass Filter	Fluorophore	Antigen	Clone	Vendor	Cat	Mix	Dilution
1	355 nm	379/28	BUV395	CD8	RPA-T8	BD Bioscience	563795	Surface	1: 80
2	65 mW	450/50	UV Blue Live/Dead	Amine Reactive	-	Invitrogen	L34962	Live/Dead	1: 500
3		515/30	BUV496	CD3	UCHT1	BD Bioscience	612940	Surface	1: 40
4		585/30	BUV563	CD25	2A3	BD Bioscience	612918	Surface	1: 40
		610/20	-	-	-	-	-	-	- -
5		670/30	BUV661	HLA-DR	G46-6	BD Bioscience	565073	Surface	1: 80
6		740/35	BUV737	ICOS	DX29	BD Bioscience	749665	Surface	1: 10
7		820/60	BUV805	CD45	HI30	BD Bioscience	612891	Surface	1: 80
8	406 nm	431/28	BV421	MR1-Tet (5-OP-RU)	-	NIH Tetramer Core	-	Pre-Surface	1: 500
9	200 mW	525/50	BV480	CD28	CD28.2	BD Bioscience	566110	Surface	1: 40
10		586/15	BV570	CD45RA	HI100	BioLegend	304132	Surface	1: 160
11		610/20	BV605	PD1	EH12.1	BD Bioscience	563245	Surface	1: 20
12		670/30	BV650	CD69	FN50	BD Bioscience	563835	Surface	1: 20
13		710/50	BV711	OX40	ACT35	BD Bioscience	563664	Surface	1: 40
14		750/30	BV750	CD103	Ber-ACT8	BD Bioscience	747099	Surface	1: 160
15		780/60	BV786	CD127	HIL-7R-M21	BD Bioscience	563324	Surface	1: 10
	488 nm	488/10	SSC	-	-	-	-		- -
16	200 mW	515/20	BB515	TIM3	7D3	BD Bioscience	565568	Surface	1: 80
17		610/20	BB630	BTLA	J168-540	BD Bioscience	2284103	Surface	1: 80
18		670/30	BB660	CD27	M-T271	BD Bioscience	624295	Surface	1: 160
19		710/50	BB700	CD161	DX12	BD Bioscience	745791	Surface	1: 20
		750/30	-	-	-	-	-	-	- -
20		780/60	BB790	CD38	HIT2	BD Bioscience	624296	Surface	1: 160
21	552 nm	586/15	PE	CD223	T47-530	BD Bioscience	565616	Surface	1: 20
22	150 mW	610/20	PE-CF594	TCRgd	B1	BD Bioscience	562511	Pre-Surface	1: 20
23		670/30	PE-Cy5	CD137	4B4-1	BD Bioscience	551137	Surface	1: 20
24		710/50	PE-Cy5.5	CD19	SJ25-C1	Invitrogen	MHCD1918	Surface	1: 160
25		780/60	PE-Cy7	CCR7	3D12	BD Bioscience	557648	Surface	1: 40
26	628 nm	670/30	eF660	KI67	SolA15	Invitrogen	50-5698-82	Intracellular	1: 500
27	200 mW	710/50	AF700	Granzyme B	GB11	BD Bioscience	560213	Intracellular	1: 80
28		780/60	APC-H7	CD4	RPA-T4	BD Bioscience	560158	Surface	1: 80

Panel 2: APC Panel

	Laser	Bandpass Filter	Fluorophore	Antigen	Clone	Vendor	Cat	Mix	Dilution
1	355 nm	379/28	BUV395	CD40	5C3	BD Bioscience	565202	Surface	1: 40
2	65 mW	450/50	UV Blue Live/Dead	Amine Reactive	-	Invitrogen	L34962	Live/Dead	1: 500
3		515/30	BUV496	CD16	3G8	BD Bioscience	612944	Surface	1: 160
4		585/30	BUV563	CD56	NCAM16.2	BD Bioscience	612928	Surface	1: 160
		610/20	-	-	-	-	-	-	- -
5		670/30	BUV661	CD3	UCHT1	BD Bioscience	612964	Surface	1: 80
6		740/35	BUV737	CD86	FUN-1	BD Bioscience	612785	Surface	1: 40
7		820/60	BUV805	CD45	HI30	BD Bioscience	612891	Surface	1: 80
8	406 nm	431/28	BV421	PD-L2	MIH18	BD Bioscience	563842	Surface	1: 20
9	200 mW	525/50	BV480	CD85k	ZM3.8	BD Bioscience	746718	Surface	1: 40
10		586/15	BV570	CD14	M5E2	BioLegend	301832	Surface	1: 20
11		610/20	BV605	CD141	1A4	BD Bioscience	740421	Surface	1: 640
12		670/30	BV650	Sirpa	SE5A5	BD Bioscience	743565	Surface	1: 160
13		710/50	BV711	CD68	Y1/82A	BD Bioscience	565594	Intracellular	1: 40
14		750/30	BV750	CD11b	ICRF44	BD Bioscience	747357	Surface	1: 80
15		780/60	BV786	CD123	7G3	BD Bioscience	564196	Surface	1: 40
	488 nm	488/10	SSC	-	-	-	-	-	- -
16	200 mW	515/20	BB515	CD206	19.2	BD Bioscience	564668	Surface	1: 20
17		610/20	BB630	BTLA	J168-540	BD Bioscience	2284103	Surface	1: 80
18		670/30	BB660	PD-L1	MIH1	BD Bioscience	624295	Surface	1: 40
19		710/50	BB700	CD32	FLI8.26	BD Bioscience	742216	Surface	1: 160
		750/30	-	-	-	-	-	-	- -
20		780/60	BB790	CD38	HIT2	BD Bioscience	624296	Surface	1: 80
21	552 nm	586/15	PE	Axl	108724	R&D	FAB154P	Surface	1: 20
*21	150 mW	586/15	PE	PDPN	NC-08	BioLegend	337003	Surface	1: 1280
22		610/20	PE-CF594	CD163	GHI/61	BD Bioscience	562670	Surface	1: 40
23		670/30	PE-Cy5	CD80	L37.4	BD Bioscience	559370	Surface	1: 10
24		710/50	PE-Cy5.5	CD19	SJ25-C1	Invitrogen	MHCD1918	Surface	1: 160
25		780/60	PE-Cy7	CX3CR1	2A9-1	BioLegend	341612	Pre-Surface	1: 160
26	628 nm	670/30	AF647	CD1c	F10/21A3	BD Bioscience	565048	Surface	1: 160
27	200 mW	710/50	AF700	CD11c	B-Ly6	BD Bioscience	561352	Surface	1: 320
28		780/60	APC-H7	HLA-DR	G46-6	BD Bioscience	561358	Surface	1: 80

*PE-PDPN instead of PE-Axl was used in longitudinal APC panel for only **Figure 6**.

Supplementary Table 2

Meta data for participant samples

Donor	Group	Draw Date Time	Received Date Time	Transit Time	Transit Hours	Transit Days	Transit Days Bin	Min Transit Temp	Max Transit Temp	Mean Transit Temp
Tasso ID 05	Vein PBMC	2022-09-26T11:39:00Z	NA	NA	NA	NA	NA	NA	NA	NA
Tasso ID 05	Vein ACK	2022-09-26T11:39:00Z	NA	NA	NA	NA	NA	NA	NA	NA
Tasso ID 05	Tasso ACK	2022-09-26T11:49:00Z	NA	NA	NA	NA	NA	NA	NA	NA
Tasso ID 05	Tasso ACK Shipped	2022-09-26T16:09:00Z	2022-09-27T12:00:00Z	0.83	19.85	0.83	0	53	77	63.50
Tasso ID 06	Vein PBMC	2022-09-19T08:29:00Z	NA	NA	NA	NA	NA	NA	NA	NA
Tasso ID 06	Vein ACK	2022-09-19T08:29:00Z	NA	NA	NA	NA	NA	NA	NA	NA
Tasso ID 06	Tasso ACK	2022-09-19T08:34:00Z	NA	NA	NA	NA	NA	NA	NA	NA
Tasso ID 06	Tasso ACK Shipped	2022-09-19T14:30:00Z	2022-09-21T09:30:00Z	1.79	43	1.79	1	50	77	63.19
Tasso ID 07	Vein PBMC	2022-09-27T11:20:00Z	NA	NA	NA	NA	NA	NA	NA	NA
Tasso ID 07	Vein ACK	2022-09-27T11:20:00Z	NA	NA	NA	NA	NA	NA	NA	NA
Tasso ID 07	Tasso ACK	2022-09-27T11:30:00Z	NA	NA	NA	NA	NA	NA	NA	NA
Tasso ID 07	Tasso ACK Shipped	2022-09-28T11:10:00Z	2022-10-03T10:00:00Z	4.95	118.83	4.95	4	52	77	62.21
Tasso ID 08	Vein PBMC	2022-09-20T09:10:00Z	NA	NA	NA	NA	NA	NA	NA	NA
Tasso ID 08	Vein ACK	2022-09-20T09:10:00Z	NA	NA	NA	NA	NA	NA	NA	NA
Tasso ID 08	Tasso ACK	2022-09-20T09:01:00Z	NA	NA	NA	NA	NA	NA	NA	NA
Tasso ID 08	Tasso ACK Shipped	2022-09-20T18:01:00Z	2022-09-26T10:00:00Z	5.67	135.98	5.67	5	50	76	62.49
Tasso ID 09	Vein PBMC	2022-09-12T08:39:00Z	NA	NA	NA	NA	NA	NA	NA	NA
Tasso ID 09	Vein ACK	2022-09-12T08:39:00Z	NA	NA	NA	NA	NA	NA	NA	NA
Tasso ID 09	Tasso ACK	2022-09-12T08:46:00Z	NA	NA	NA	NA	NA	NA	NA	NA
Tasso ID 09	Tasso ACK Shipped	2022-09-12T17:28:00Z	2022-09-13T11:30:00Z	0.75	18.03	0.75	0	62	70	64.61
Tasso ID 10	Vein PBMC	2022-09-19T09:23:00Z	NA	NA	NA	NA	NA	NA	NA	NA
Tasso ID 10	Vein ACK	2022-09-19T09:23:00Z	NA	NA	NA	NA	NA	NA	NA	NA
Tasso ID 10	Tasso ACK	2022-09-19T09:28:00Z	NA	NA	NA	NA	NA	NA	NA	NA
Tasso ID 10	Tasso ACK Shipped	2022-09-19T17:30:00Z	2022-09-21T09:30:00Z	1.67	40	1.67	1	50	77	62.43
Tasso ID 11	Vein PBMC	2022-09-06T08:03:00Z	NA	NA	NA	NA	NA	NA	NA	NA
Tasso ID 11	Vein ACK	2022-09-06T08:03:00Z	NA	NA	NA	NA	NA	NA	NA	NA
Tasso ID 11	Tasso ACK	2022-09-06T08:09:00Z	NA	NA	NA	NA	NA	NA	NA	NA
Tasso ID 11	Tasso ACK Shipped	2022-09-06T17:17:00Z	2022-09-09T11:00:00Z	2.74	65.72	2.74	2	52	76	64.21
Tasso ID 12	Vein PBMC	2022-09-26T11:53:00Z	NA	NA	NA	NA	NA	NA	NA	NA
Tasso ID 12	Vein ACK	2022-09-26T11:53:00Z	NA	NA	NA	NA	NA	NA	NA	NA
Tasso ID 12	Tasso ACK	2022-09-26T12:07:00Z	NA	NA	NA	NA	NA	NA	NA	NA
Tasso ID 12	Tasso ACK Shipped	2022-09-26T19:19:00Z	2022-09-28T10:00:00Z	1.61	38.68	1.61	1	53	77	61.40
Tasso ID 13	Vein PBMC	2022-09-12T10:52:00Z	NA	NA	NA	NA	NA	NA	NA	NA
Tasso ID 13	Vein ACK	2022-09-12T10:52:00Z	NA	NA	NA	NA	NA	NA	NA	NA
Tasso ID 13	Tasso ACK	2022-09-12T10:59:00Z	NA	NA	NA	NA	NA	NA	NA	NA
Tasso ID 13	Tasso ACK Shipped	2022-09-12T13:31:00Z	2022-09-14T11:00:00Z	1.9	45.48	1.9	1	62	73	65.56
Tasso ID 14	Vein PBMC	2022-09-19T11:16:00Z	NA	NA	NA	NA	NA	NA	NA	NA
Tasso ID 14	Vein ACK	2022-09-19T11:16:00Z	NA	NA	NA	NA	NA	NA	NA	NA
Tasso ID 14	Tasso ACK	2022-09-19T11:23:00Z	NA	NA	NA	NA	NA	NA	NA	NA
Tasso ID 14	Tasso ACK Shipped	2022-09-19T17:51:00Z	2022-09-22T10:00:00Z	2.67	64.15	2.67	2	50	77	63.00
Tasso ID 15	Vein PBMC	2022-09-06T11:03:00Z	NA	NA	NA	NA	NA	NA	NA	NA
Tasso ID 15	Vein ACK	2022-09-06T11:03:00Z	NA	NA	NA	NA	NA	NA	NA	NA
Tasso ID 15	Tasso ACK	2022-09-06T11:26:00Z	NA	NA	NA	NA	NA	NA	NA	NA
Tasso ID 15	Tasso ACK Shipped	2022-09-06T16:30:00Z	2022-09-08T10:21:00Z	1.74	41.85	1.74	1	52	76	64.95
Tasso ID 17	Vein PBMC	2022-09-20T08:23:00Z	NA	NA	NA	NA	NA	NA	NA	NA
Tasso ID 17	Vein ACK	2022-09-20T08:23:00Z	NA	NA	NA	NA	NA	NA	NA	NA
Tasso ID 17	Tasso ACK	2022-09-20T08:39:00Z	NA	NA	NA	NA	NA	NA	NA	NA
Tasso ID 17	Tasso ACK Shipped	2022-09-20T11:40:00Z	2022-09-21T09:30:00Z	0.91	21.83	0.91	0	50	77	63.32
Tasso ID 18	Vein PBMC	2022-09-27T08:45:00Z	NA	NA	NA	NA	NA	NA	NA	NA
Tasso ID 18	Vein ACK	2022-09-27T08:45:00Z	NA	NA	NA	NA	NA	NA	NA	NA
Tasso ID 18	Tasso ACK	2022-09-27T08:53:00Z	NA	NA	NA	NA	NA	NA	NA	NA
Tasso ID 18	Tasso ACK Shipped	2022-09-27T13:08:00Z	2022-09-29T10:00:00Z	1.87	44.87	1.87	1	55	77	60.69
Tasso ID 19	Vein PBMC	2022-09-19T10:00:00Z	NA	NA	NA	NA	NA	NA	NA	NA
Tasso ID 19	Vein ACK	2022-09-19T10:00:00Z	NA	NA	NA	NA	NA	NA	NA	NA
Tasso ID 19	Tasso ACK	2022-09-19T10:07:00Z	NA	NA	NA	NA	NA	NA	NA	NA
Tasso ID 19	Tasso ACK Shipped	2022-09-19T13:56:00Z	2022-09-21T09:30:00Z	1.82	43.57	1.82	1	50	77	63.19
Tasso ID 20	Vein PBMC	2022-09-20T09:06:00Z	NA	NA	NA	NA	NA	NA	NA	NA
Tasso ID 20	Vein ACK	2022-09-20T09:06:00Z	NA	NA	NA	NA	NA	NA	NA	NA
Tasso ID 20	Tasso ACK	2022-09-20T09:15:00Z	NA	NA	NA	NA	NA	NA	NA	NA
Tasso ID 20	Tasso ACK Shipped	2022-09-21T07:30:00Z	2022-09-22T10:00:00Z	1.1	26.5	1.1	1	55	76	63.54
Tasso ID 21	Vein PBMC	2022-09-27T10:37:00Z	NA	NA	NA	NA	NA	NA	NA	NA
Tasso ID 21	Vein ACK	2022-09-27T10:37:00Z	NA	NA	NA	NA	NA	NA	NA	NA
Tasso ID 21	Tasso ACK	2022-09-27T10:45:00Z	NA	NA	NA	NA	NA	NA	NA	NA
Tasso ID 21	Tasso ACK Shipped	2022-09-27T19:03:00Z	2022-09-29T10:00:00Z	1.62	38.95	1.62	1	55	65	59.02

Supplementary Table 3

Meta data for longitudinal replicates

	Date of Staining	Date of Acquisition	Biologic Replicate File Name	Rainbow Beads File Name
*0	NA	2023-03-08	NA	rainbow beads_2023-03-08 New Lot AP03.fcs
1	2023-08-22	2023-08-24	TUM209_BP0323502.fcs	rainbow beads_2023-08-24 Lot AP03.fcs
2	2023-08-23	2023-08-24	TUM210_BP0323502.fcs	
3	2023-09-11	2023-09-13	TUM212_BP0323502.fcs	rainbow beads_2023-09-13 Lot AP03.fcs
4	2023-09-12	2023-09-13	TUM213_BP0323502.fcs	
5	2023-10-11	2023-10-12	TUM215_BP0323502.fcs	rainbow beads_2023-10-12 Lot AP03.fcs
6	2023-10-23	2023-10-24	TUM218_BP0323502.fcs	rainbow beads_2023-10-24 AP03.fcs
7	2023-11-01	2023-11-06	TUM219_BP0323502.fcs	rainbow beads_2023-11-06 Lot AP03.fcs
8	2024-01-29	2024-01-31	mfPLAC_BP0323502.fcs	rainbow beads_2024-01-31 Lot AP03.fcs
9	2024-08-28	2024-08-30	TUM225_BP0323502.fcs	rainbow beads_2024-08-30 Lot AP03.fcs
10	2025-04-29	2025-04-30	Frozen_BP0323502.fcs	rainbow beads_2025-04-30 Lot AP03.fcs

* Calibration of rainbow beads lot AP03, no biological samples stained
